# In memoriam: Konjev Desender (1956–2008) and Jean-Pierre Maelfait (1951–2009)

**DOI:** 10.3897/zookeys.100.1520

**Published:** 2011-05-20

**Authors:** Gábor L. Lövei

**Affiliations:** Aarhus University, Faculty of Agricultural Sciences, Department of Integrated Pest Management, Flakkebjerg Research Centre, DK-4200 Slagelse, Denmark

It is about a year ago that Belgian biology lost, in quick sequence, two distinguished and internationally known representatives: Konjev Desender and Jean-Pierre Maelfait.

Before contemplating this sad loss, allow me to mention some facts, even if they are well known in these circles. Konjev Desender was born in 1956, and graduated in 1978 from the University of Ghent. In 1987, he received his PhD, and in 1990, he joined the Belgian Institute of Natural Sciences, where he remained until his untimely death from cancer at the age of 52. Originally interested in birds, Konjev’s attention later turned to ground beetles, and after 1980, he mainly worked with invertebrates. The focus of his research was ground beetles, especially in relation to nature conservation, forestry, and population genetics. He took part in 6 expeditions to the Galapagos Islands (for which I envied him a great deal), has worked on salt marshes around Europe, but above all, worked and collected intensively in Belgium. His work was massive in terms of numbers of beetles collected, driven by keen curiosity, and he understood well that human activities have shaped even the invertebrate fauna of his country. I really liked his work on identifying beetle remains in old wells excavated by archeologists, giving a richer understanding of environments past. This was, however just one facet of his varied activities. He was a hard worker, and published over 350 papers, mostly in collaboration, guided numerous younger scientists, and was active in the Belgian entomological society. He also organised one of the European carabidologist meetings, in 1992, in collaboration with his friend Jean-Pierre Maelfait and other Belgian colleagues.

I have first met both of them, I believe, at a soil zoology symposium in Louvain-la-Neuve in 1982, and recall the intensity in Konjev’s voice when he talked about his research, and the quiet presence of Jean-Pierre.

I remember Konjev mostly through our encounters at meetings like this one, where he has always been present (actually more frequently than myself), and I do not think there are many in this room who do not remember him as one of the central figures of our carabidologist meetings.

Jean-Pierre was a more relaxed, one could even say slower, but no less impressive personality. Jean-Pierre Maelfait was born in 1951, 5 years senior to Konjev, and spent most of his career at the University of Ghent and at the Institute for Nature Conservation and Forestry in Brussels (INBO). Like for many others who graduated from that university, he was also Konjev’s teacher, later a colleague and friend. As a teacher, he will be long remembered – I believe there are several participants in this room who were Jean-Pierre’s students, and I am sure he has made a profund influence on your knowledge and professional attitude. Jean-Pierre was also a productive scientist – his output, some of which is still being published, amounts to ca. 300 items. These include numerous articles on ground beetles, but his speciality was arachnology, the venerable study of spiders. Jean-Pierre and Konjev shared a similar approach to science and nature, were very active in the conservation of their respective groups, and also their habitats; they also shared several projects. Both of them visited the Galapagos Islands several times, and published on their results from there over a period of more than a decade. Their first shared publications go back to 1980; altogether they are co-authors on 100 published papers. These concern surface-active organisms, papers on the inhabitants of Belgian sand dunes, forest invertebrates, ecological restoration, and so on.

Due to this shared interest and close relationship, Jean-Pierre was originally asked by the organisers of this meeting to talk about Konjev here. Then, suddenly and unexpectedly, he died. He was 57; hardly older than Konjev. And this is how this mantle, this not-so-light mantle fell on me.

**Figure:**
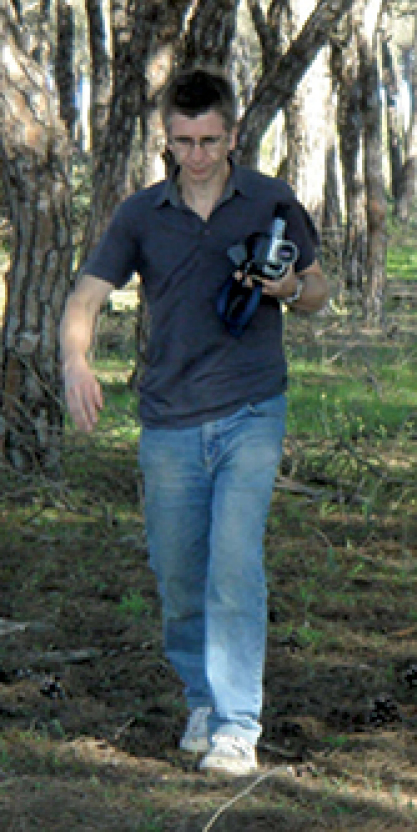
Konjev Desender

**Figure:**
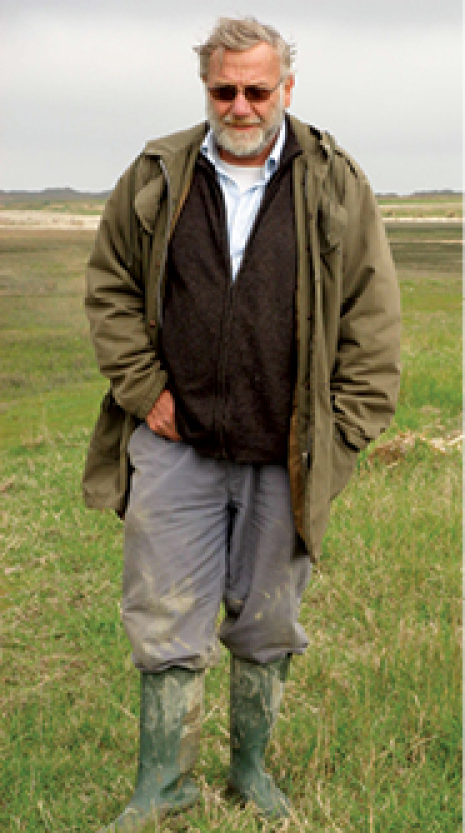
Jean-Pierre Maelfait

I have had a very dear New Zealand friend, sadly also dead, by the name of John Bevan Ford. He was a respected and much loved New Zealand Maori artist. Once he was explaining to me the Maori attitude to life, its continuity and death. When someone dies, he said, people who knew him gather together to celebrate his or her life, and talk to him as someone still much present, even if unable to answer. If they had a quarrel, they mention it. If they have unsorted business, they lament that this can now never be laid to rest. And they mention how much they enjoyed when they did things together, and rejoice again. In this spirit I would like to remember these two colleagues of us. I think it is wise and very fitting. I, for one, can now never berate Konjev and convince him that one does not need to kill tens of thousands of beetles to illustrate an ecological phenomenon. He will not kill more beetles now, but I am not happy. And I smiled again, when I saw the photos of Konjev and Johan Kotze, at the conference party at Mols, Denmark, playing with empty beer glasses, and a few coins. That photo is so full of the joy of life...I am sure that we all carry similar memories of both of them.

So when we rember them now, I do not ask you to stand up and think silently about them, to show respect. Respect we have for them and respect we will continue to show them. But I would also like to quote a Japanese haiku, which says that a sumo wrestler shows respect for his teacher by winning over him on the wrestling mat. Science is not about fighting, but this, in a way, we shall also do – to build on their work, and proceed further. I know that they had no intention to stop and see only us advance, nor would they have withdrawn their support when younger colleagues were preparing to surpass them. We are only a little sad that they themselves were stopped.

Bearing such a loss is not easy. When I now turn to the families of Jean-Pierre and Konjev, I know that these words do not bring them back, and hearing these words may make their grief swell again. Jean-Pierre will not again silently, gently sit among us, and Konjev’s sharp voice will not be heard again at future meetings. Nevertheless, I would still like, in the name of all of us, to say to you, that we are glad. We are glad to have known them, even if we met them only sporadically, and we thank you for providing support for them. None of us can function without this support. We, carabidologists are an odd race, in finding small, smelly creatures of strange habit fascinating, interesting, and well worth spending days (months) away from home, coming home late, and going into our workplace or field site even on weekends. This is all time taken from you, from our family. We mostly cannot help it and we can only ask for understanding. We are probably not party stars when it comes to our work, and I cannot imagine people with open mouths and jealous expressions when it comes to mentioning our work with carabids; yet we are always willing to talk about them. We hope you understood that neither Konjev, nor Jean-Pierre was odd nor slightly crazy – and hope you believe we are not, either. I hope both of them found time for you as well as carabids, spiders, and work, and that they were appropriate support and good company. Whatever they did in science, at the last count is not so important. If they managed to instill in their children, friends, and family, that life on Earth is wonderful, and studying it is worth spending a life with, then you, or we, cannot ask for more.

In closing this short remembrance, allow me to voice my gratitude that I, that we, have had Jean-Pierre and Konjev in our lives. I would like to thank the organisers for thinking about this remembrance which I feel is appropriate, and thank you for bearing with me. I hope the families of Jean-Pierre and Konjev will find some consolation to know how much we appreciated these two colleagues of ours as scientsts and human beings. May all of us, when the time comes, have left as rich and inspiring a legacy as Konjev and Jean-Pierre did, and be remembered this fondly.

Thank you.

## Bibliographical List

This list has been compiled by Konjev and Jean-Pierre's colleague and initiator of the Galapagos project, Dr. Léon Baert of the Belgian Institute of Natural Sciences (KBIN), Vautierstraat 29, 1000 Bruxelles.

1. DESENDER K (1979) Het Blankaartgebied. De Gidsenkring, 17: 2-9.

2. DESENDER K, HOUWEN P (1979) De cetti’s zanger in het Blankaartreservaat. De Wielewaal, 45: 144-146.

3. DESENDER K (1980) Historiek van de belangrijkste reigerbroedplaatsen in de Westhoek. De Gidsenkring, 18: 21-26.

4. DESENDER K (1980) Watervogels in het Blankaartbekken tijdens het winterhalfjaar. Natuurreservaten (Bulletin B.N.V.R.), 27: 54-57.

5. DESENDER K (1980) Les oiseaux d’eau du bassin du Blankaart en hiver. Réserves Naturelles (Bulletin R.N.O.B.), 27: 54-57.

6. DESENDER K, HOUWEN P (1980) Bruine kiekendieven (*Circus aeruginosus*) op de Blankaart. De Wielewaal, 46: 437-442.

7. DESENDER K, MAELFAIT J-P, DEURINCK R (1980) Ecological data on *Trechus obtusus* De Geer (Coleoptera, Carabidae) collected by pitfall trapping in coastal dunes (Belgium). Biologisch Jaarboek Dodonaea, 48: 90-101.

8. DESENDER K, MAELFAIT J-P, VANHERCKE L, DEURINCK R (1980) Investigations on Coleoptera communities in different habitats. I. The carabid fauna of the dune nature reserve ‘De Westhoek’ (De Panne, Belgium). Biologisch Jaarboek Dodonaea, 48: 102-110.

9. MAELFAIT J-P, DESENDER K, STEENHOUDT R, VANHERCKE L (1980) Coexistence of carabid beetles. Biologisch Jaarboek Dodonaea, 48: 119-125.

10. VANHERCKE L, MAELFAIT J-P, DESENDER K (1980) Beetles captured by means of a light trap. Biologisch Jaarboek Dodonaea, 48: 153-162.

11. DESENDER K (1981) Informatie en wenken voor strandonderzoek. De Strandvlo, 1: 4-8.

12. DESENDER K (1981) Garnalen, kreeften en krabben (Decapoda) langs de Westkust. De Strandvlo, 1: 32-37.

13. DESENDER K (1981) Heremietkreeften en hun parasieten. De Strandvlo, 1: 60-64.

14. DESENDER K (1981) Aantekeningen over drie interessante kreeftachtigen (Crustacea, Decapoda). De Strandvlo, 1: 72-75.

15. DESENDER K (1981) Uit het Natuurhistorisch Archief. Aflevering 2. De Strandvlo, 1: 110-114.

16. DESENDER K, MAELFAIT J-P, D’HULSTER M, VANHERCKE L (1981) Ecological and faunal studies on Coleoptera in agricultural land. I. Seasonal occurrence of Carabidae in the grassy edge of a pasture. Pedobiologia, 22: 379-384.

17. MAELFAIT J-P, VANHERCKE L, DESENDER K (1981) Coleopterologische mededelingen van het Laboratorium voor Oecologie der Dieren, Zoögeografie en Natuurbehoud (R.U.G.). I. Bulletin et Annales de la Société royale belge d’Entomologie, 117: 84-85.

18. DESENDER K, VANHERCKE L, MAELFAIT J-P (1981) Coleopterologische mededelingen van het Laboratorium voor Oecologie der Dieren, Zoögeografie en Natuurbehoud (R.U.G.). II. Bulletin et Annales de la Société royale belge d’Entomologie, 117: 164-165.

19. RAPPE G, DESENDER K (1981) Eikapsels van haaien en roggen langs de Belgische kust, een eerste bericht. De Strandvlo, 1: 65-71.

20. DESENDER K (1982) Over het meten van eikapsels van haaien en roggen. De Strandvlo, 2: 16-20.

21. DESENDER K (1982) Ecological and faunal studies on Coleoptera in agricultural land. II. Hibernation of Carabidae in agro-ecosystems. Pedobiologia, 23: 295-303.

22. DESENDER K (1982) Eenden in het Blankaartbekken. Overzicht van de watervogeltellingen tijdens de vier laatste winters (1977-1981). De Wielewaal, 48: 49-53.

23. DESENDER K, D’HULSTER M, MAELFAIT J-P, VANHERCKE L (1982) Review 3. Onderzoek uit het Laboratorium voor Oecologie der Dieren, Zoögeografie en Natuurbehoud. Oecologie van weide-Arthropoda. Biologisch Jaarboek Dodonaea, 50: 45-50.

24. DESENDER K, HUBLE J, VANHERCKE L (1982) Loopkevers, spinnen en hooiwagens van het duinreservaat ‘De Kijkuit’ te De Haan (West-Vlaanderen). Phegea, 10: 201-214.

25. DESENDER K, MAELFAIT J-P, VANHERCKE L (1982) Variations qualitatives saisonnières des Carabidae (Coleoptera) d’une prairie pâturée à Melle (Flandre Orientale, Belgique), étudiés à l’aide de différentes méthodes d’échantillonnage. Biologisch Jaarboek Dodonaea, 50: 83-92.

26. D’HULSTER M, DESENDER K (1982) Ecological and faunal studies on Coleoptera in agricultural land. III. Seasonal abundance and hibernation of Staphylinidae in the grassy edge of a pasture. Pedobiologia, 23: 403-414.

27. DESENDER K (1983) Ecological data on *Clivina fossor* (Coleoptera, Carabidae) from a pasture ecosystem. 1. Adult and larval abundance, seasonal and diurnal activity. Pedobiologia, 25: 157-167.

28. DESENDER K (1983) *Trechus rivularis* Belg. n. sp. (Coleoptera, Carabidae), une espèce subarctique des Hautes Fagnes (Mont Rigi, Belgique). Bulletin et Annales de la Société royale belge d’Entomologie, 119: 177-183.

29. DESENDER K (1983) De Aalscholver *Phalacrocorax carbo sinensis* in het Blankaartgebied. De Wielewaal, 49: 421-424.

30. DESENDER K (1983) Loopkevers van het natuurreservaat ‘De Maten’ te Genk (Limburg) (Coleoptera, Carabidae). Phegea, 11: 49-54.

31. DESENDER K, CRAPPE D (1983) Larval and adult morphology and biometry of two sibling species *Bembidion lampros* (Herbst) and *Bembidion properans* Stephens (Coleoptera, Carabidae). Biologisch Jaarboek Dodonaea, 51: 36-54.

32. VANHERCKE L, DESENDER K, MAELFAIT J-P (1983) Coleopterologische mededelingen van het Laboratorium voor Oecologie de Dieren, Zoögeografie en Natuurbehoud (R.U.G.). III. Bulletin et Annales de la Société royale belge d’Entomologie, 119: 52-54.

33. DESENDER, K., D’HULSTER, M., MAELFAIT, J.-P., VANHERCKE, L., 1983. Coleopterologische mededelingen van het Laboratorium voor Oecologie der Dieren, Zoögeografie en Natuurbehoud (R.U.G.). IV. Bulletin et Annales de la Société royale belge d’Entomologie, 119: 283-285.

34. DESENDER, K., MAELFAIT, J.-P., 1983.. Population restoration by means of dispersal, studied for different carabid beetles (Coleoptera, Carabidae) in a pasture ecosystem. In: LEBRUN, Ph., ANDRE, H.M., DE MEDTS, A., GREGOIRE-WIBO, C., WAUTHY, G. (eds.). New Trends in Soil Biology. Proceedings of the VIII. International Colloquium on Soil Zoology, Louvain-la-Neuve (Belgium), August 30 - September 2, 1982. Dieu-Brichart, Ottignies-Louvain-la-Neuve, 541-550.

35. DESENDER, K., PANNE, V., 1983. The larvae of *Pterostichus strenuus* Panzer and *Pterostichus vernalis* Panzer (Coleoptera, Carabidae). Annales de la Société royale zoologique de Belgique, 113: 139-154.

36. DESENDER, K., POLLET, M., MAELFAIT, J.-P., 1983. Coleopterologische mededelingen van het Laboratorium voor Oecologie der Dieren, Zoögeografie en Natuurbehoud (R.U.G.). V. Carabidae (Coleoptera) in het staatsnatuurreservaat ‘Ten Haagdoornheide’. Bulletin et Annales de la Société royale belge d’Entomologie, 119: 321-322.

37. DESENDER, K. 1983. Curlew (*Numenius arquata*) food preference on an inland roosting place during winter. Le Gerfaut, 73: 407-409.

38. DESENDER, K., 1984. Faunistiek van loopkevers in België. De loopkevers van de collectie Hostie (Coleoptera, Carabidae). Phegea, 12: 1-12.

39. DESENDER, K., 1984. De betekenis en het gebruik van bodemvallen voor faunistisch- oecologisch onderzoek van bodemoppervlakteaktieve ongewervelden. Phegea, 12: 85-94.

40. DESENDER, K., HUYSSEUNE, A., 1984. Aantekeningen over Chinese wolhandkrabben. Het Zeepaard, 43: 198-204.

41. DESENDER, K., HOUWEN, P., 1984. Voedseltrek van eenden - slaaptrek van meeuwen. Verslag van een gedetailleerde avondtelling in het Blankaartgebied. De Wielewaal, 50: 143-149.

42. DESENDER, K., MERTENS, J., D’HULSTER, M., BERBIERS, P., 1984. Diel activity patterns of Carabidae (Coleoptera), Staphylinidae (Coleoptera) and Collembola in a heavily grazed pasture. Revue d’Ecologie et Biologie du Sol, 21: 347-361.

43. DESENDER, K., VANEECHOUTTE, M., 1984. Phoretic associations of carabid beetles (Coleoptera, Carabidae) and mites (Acari). Revue d’Ecologie et Biologie du Sol, 21: 363-371.

44. D’HULSTER, M., DESENDER, K., 1984. Ecological and faunal studies of Coleoptera in agricultural land. IV. Hibernation of Staphylinidae in agro-ecosystems. Pedobiologia, 26: 65-73.

45. POLLET, M., DESENDER, K., MAELFAIT, J.-P., 1984. Coleopterologische mededelingen van het Laboratorium voor Oecologie der Dieren, Zoögeografie en Natuurbehoud (R.U.G.). VI. Carabidae (Coleoptera) van Veldegem (West-Vlaanderen). Bulletin et Annales de la Société royale belge d’Entomologie, 120: 321-325.

46. DESENDER, K., MAELFAIT, J.-P., POLLET, M., 1984. Coleopterologische mededelingen van het Laboratorium voor Oecologie der Dieren, Zoögeografie en Natuurbehoud (R.U.G.). VII. Carabidae (Coleoptera) in het natuurreservaat ‘De Fonteintjes’ (Blankenberge) en duinbosjes te De Haan (West-Vlaanderen). Bulletin et Annales de la Société royale belge d’Entomologie, 120: 320-342.

47. DESENDER, K., POLLET, M., 1984. Captures remarquables de Coléoptères Carabiques en Gaume. Bulletin et Annales de la Société royale belge d’Entomologie, 120: 342-351.

48. DESENDER, K., POLLET, M., MAELFAIT, J.-P., 1984. Faunistiek van loopkevers in ons land - een eerste balans. Bulletin et Annales de la Société royale belge d’Entomologie, 120: 353-355.

49. SEGERS, H., DESENDER, K., ANSELIN, A., 1984. Coleopterologische mededelingen van het Laboratorium voor Oecologie der Dieren, Zoögeografie en Natuurbehoud (R.U.G.). VIII. Staphylinidae (Coleoptera) in het Oost-Vlaamse Krekengebied. Bulletin et Annales de la Société royale belge d’Entomologie, 120: 371-375.

50. DESENDER, K., POLLET, M., SEGERS, H., 1984. Carabid beetle distribution along humidity-gradients in rivulet-associated grasslands (Coleoptera, Carabidae). Biologisch Jaarboek Dodonaea, 52: 64-75.

51. DESENDER, K., 1985. Faunistiek van loopkevers in België. II. De loopkevers van de collectie DE RIE (Coleoptera: Carabidae). Phegea, 13: 21-28.

52. DESENDER, K., 1985. Naamlijst van de loopkevers en zandloopkevers van België (Coleoptera, Carabidae). Studiedocumenten Nr 19, Koninklijk Belgisch Instituut voor Natuurwetenschappen, Brussel, 36 pp.

53. DESENDER, K., 1985. Liste des espèces de Carabes et de Cicindèles de Belgique (Coleoptera, Carabidae). Documents de travail N° 21, Institut Royal des Sciences naturelles de belgique, Bruxelles, 36 pp.

54. DESENDER, K., 1985. Graslandbeheer en invertebraten. Natuurreservaten, 7: 88-91.

55. DESENDER, K., POLLET, M., 1985. Ecological data on *Clivina fossor* (Coleoptera, Carabidae) from a pasture ecosystem. II. Reproduction, biometry, biomass, wing polymorphism and feeding ecology. Revue d’Ecologie et Biologie du Sol, 22: 233-246.

56. DESENDER, K., POLLET, M., 1985. Coleopterologische mededelingen van het Laboratorium voor Oecologie der Dieren, Zoögeografie en Natuurbehoud (R.U.G.). X. Carabidae (Coleoptera) van Heure-en-Famenne en omgeving. Bulletin et Annales de la Société royale belge d’Entomologie, 121: 48.

57. DESENDER, K., ANSELIN, A., SEGERS, H., 1985. Coleopterologische mededelingen van het Laboratorium voor Oecologie der Dieren, Zoögeografie en Natuurbehoud (R.U.G.). IX. Carabidae (Coleoptera) in het Oost-Vlaams Krekengebied. Bulletin et Annales de la Société royale belge d’Entomologie, 121: 46-48.

58. DESENDER, K., 1985. Carabid beetles new for the belgian fauna. Bulletin et Annales de la Société royale belge d’Entomologie, 121: 69-74.

59. DESENDER, K., VAN DEN BROECK, D., MAELFAIT, J.-P., 1985. Population biology and reproduction in *Pterostichus melanarius* ILL. (Coleoptera, Carabidae) from a heavily grazed pasture ecosystem. Mededelingen van de Faculteit Landbouwwetenschappen van de Rijksuniversiteit Gent, 50: 567-575.

60. POLLET, M.,, DESENDER, K., 1985. Adult and larval feeding ecology in *Pterostichus melanarius* ILL. (Coleoptera, Carabidae). Mededelingen van de Faculteit Landbouwwetenschappen van de Rijksuniversiteit Gent, 50: 581-594.

61. DESENDER, K., 1985. Wing polymorphism and reproductive biology in the halobiont carabid beetle *Pogonus chalceus* (MARSHAM) (Coleoptera, Carabidae). Biologisch Jaarboek Dodonaea, 53: 89-100.

62. DESENDER, K., 1985. Coleopterologische mededelingen van het Laboratorium voor Oecologie der Dieren, Zoögeografie en Natuurbehoud (R.U.G.). XI. Carabidae (Coleoptera) in het staatsnatuurreservaat ‘De Kalmthoutse Heide’ (Antwerpen, België). Bulletin et Annales de la Société royale belge d’Entomologie, 121: 470-472.

63. POLLET, M., DESENDER, K., 1985. Coleopterologische mededelingen van het Laboratorium voor Oecologie der Dieren, Zoögeografie en Natuurbehoud (R.U.G.). XII. Carabidae (Coleoptera) van het Groenhovebos te Torhout (West-Vlaanderen). Bulletin et Annales de la Société royale belge d’Entomologie, 121: 48-485.

64. POLLET, M., DESENDER, K., MAELFAIT, J.-P., 1985. Onderzoek naar de voedselkeuze van loopkevers (Carabidae, Coleoptera) in weide-oecosystemen. Bulletin et Annales de la Société royale belge d’Entomologie, 121: 494-497.

65. DESENDER, K., HUYSSEUNE, A., 1985. Captures remarquables de Coléoptères Cerambycidae et Carabidae de la région du Viroinval. Bulletin et Annales de la Société royale belge d’Entomologie, 121: 503-505.

66. DESENDER, K., SEGERS, H., 1985. A simple device and technique for quantitative sampling of riparian beetle populations with some carabid and staphylinid abundance estimates on different riparian habitats (Coleoptera). Revue d’Ecologie et Biologie du Sol, 22: 497-506.

67. VAN DEN HEUVEL, R., DESENDER, K., 1986. Een merkwaardige vondst van *Bembidion stomoides* DEJEAN, 1831 (Coleoptera: Carabidae). Phegea, 14: 37-38.

68. DESENDER, K., 1986. Note sur l’extension et la distribution de *Cicindela silvicola* DEJEAN, 1822. L’Entomologiste, 42: 201-203.

69. DESENDER, K., 1986. Ecology, distribution and dispersal power of endangered Carabid beetles in Belgium. Proceedings of the 3rd European Congress of Entomology, Part 3: 429-432.

70. MERCKEN, L., DESENDER, K., BOSMANS, R., APPELS, J., 1986. Empirical test on the evaluation of the concept ‘rare species’ in waterbugs and waterbeetles. Proceedings of the 3rd European Congress of Entomology, Part 1: 182.

71. MERCKEN, L., DESENDER, K., MAELFAIT, J.-P., 1986. Characterization and ecological interpretation of morphological traits related to size and dispersal power in Corixidae and Gerridae. Proceedings of the 3rd European Congress of Entomology, Part 1: 183.

72. DESENDER, K., VAN KERCKVOORDE, M., DE KIMPE, A., MAELFAIT, J.-P., 1986. The influence of the substratum on the habitat selection of riparian Carabid beetles in Belgium. Proceedings of the 3rd European Congress of Entomology, Part 3: 524.

73. DESENDER, K., VAN KERCKVOORDE, M., MAELFAIT, J.-P., 1986. Can motorway verges and urban habitats contribute to the conservation of Carabid beetles in Belgium? Proceedings of the 3rd European Congress of Entomology, Part 3: 525.

74. POLLET, M., DESENDER, K., 1986. Habitat preference and rarity of Carabid beetles (Col., Carabidae) from different woodland sites in Western Flanders (Belgium). Proceedings of the 3rd European Congress of Entomology, Part 3: 528.

75. DESENDER, K., 1986. Distribution and ecology of Carabid beetles in Belgium (Coleoptera, Carabidae). Part 1. Species 1-80 (Cicindelini, Omophrinini, Carabini, Cychrini, Nebriini, Notiophilini, Elaphrini, Loricerini, Scaritini, Broscini, Patrobini, Trechini). Studiedocument Nr 26, Koninklijk Belgisch Instituut voor Natuurwetenschappen, Brussel, 30 pp.

76. DESENDER, K., 1986. Distribution and ecology of Carabid beetles in Belgium (Coleoptera, Carabidae). Part 2. Species 81-152 (Bembidiini, Pogonini). Studiedocument Nr 27, Koninklijk Belgisch Instituut voor Natuurwetenschappen, Brussel, 24 pp.

77. DESENDER, K., 1986. Distribution and ecology of Carabid beetles in Belgium (Coleoptera, Carabidae). Part 3. Species 153-217 (Pterostichini, Perigonini). Studiedocument Nr 30, Koninklijk Belgisch Instituut voor Natuurwetenschappen, Brussel, 23 pp.

78. DESENDER, K., 1986. Distribution and ecology of Carabid beetles in Belgium (Coleoptera, Carabidae). Part 4. Species 218-379 (Amarini, Zabrini, Harpalini, Licinini, Chlaeniini, Oodini, Panagaeini, Odacanthini, Masoreini, Lebiini, Brachinini). Studiedocument Nr 34, Koninklijk Belgisch Instituut voor Natuurwetenschappen, Brussel, 48 pp.

79. DESENDER, K., 1986. On the relation between abundance and flight activity in Carabid beetles from a heavily grazed pasture. Zeitschrift für Angewandte Entomologie, 102: 225-231.

80. DESENDER, K., MAELFAIT, J.-P., VANEECHOUTTE, M., 1986. Allometry and Evolution of Hind Wing Development in Macropterous Carabid Beetles. In: DEN BOER, P.J., LUFF, M., MOSSAKOWSKI, D, WEBER, F. (eds). Carabid beetles. Their adaptations and Dynamics. Gustav Fisher, Stuttgart, 101-112.

81. DESENDER, K., TURIN, H., 1986. Overeenkomsten en verschillen bij recente veranderingen in de samenstelling van de loopkeverfauna in vier Westeuropese landen (Coleoptera: Carabidae). Nieuwsbrief European Invertebrate Survey - Nederland, 17: 23-32.

82. DESENDER, K., POLLET, M., 1986. Adult and larval abundance from Carabid Beetles (Col., Carabidae) in a pasture under changing grazing management. Mededelingen van de Faculteit Landbouwwetenschappen van de Rijkuniversiteit Gent, 51/3a: 943-955.

83. MAELFAIT, J.-P., DESENDER, K., 1986. L’effort de reproduction chez quelques espèces de Lycosidae (Lycosidae, Araneae). Mémoires de la Société royale belge d’Entomologie, 33: 224.

84. POLLET, M., DESENDER, K., 1986. Prey selection in Carabid Beetles (Col., Carabidae): are diel activity patterns of predators and prey synchronized? Mededelingen van de Faculteit Landbouwwetenschappen van de Rijksuniversiteit Gent, 51/3a: 957-971.

85. DESENDER, K., MAELFAIT, J.-P., 1986. Pitfall trapping within enclosures: a method for estimating the relationship between the abundance of coexisting carabid species (Coleoptera, Carabidae). Holarctic Ecology, 9: 245-250.

86. MERTENS, J., DESENDER, K., VAN KERCKVOORDE, M., 1986. Insect fauna from a Roman well at Maldegem (Belgium). In: THOEN, H, VANDERMOERE, N. (NENQUIN, J., ed.). The Roman fortified Site at Maldegem (East Flanders). 1985 Excavation Report. Scholae Archaeologicae, 6: 45-48.

87. POLLET, M., DESENDER, K., 1986. Detailed distribution and faunistics of carabid beetles (Coleoptera, Carabidae) in a meadow habitat (Western Flanders). Bulletin et Annales de la Société royale belge d’Entomologie, 122: 203-211.

88. DESENDER, K., MERCKEN, L., POLLET, M., VAN KERCKVOORDE, M., 1986. Redécouverte de plusieurs Coléoptères Carabiques rares le long de la Meuse (Belgique). Bulletin et Annales de la Société royale belge d’Entomologie, 122: 279-283.

89. ALDERWEIRELDT, M., DESENDER, K., 1986. Faunistisch interessante loopkevers (Coleoptera, Carabidae) van een weide met berm te Sint-Denijs-Westrem. Bulletin et Annales de la Société royale belge d’Entomologie, 122: 303-305.

90. DESENDER, K., POLLET, M., BAERT, L., KEKENBOSCH, J., 1986. Carabidae (Coleoptera) collected with pitfall traps in different habitats at Ferrières and Xhoris (Belgium). Bulletin et Annales de la Société royale belge d’Entomologie, 122: 321-323.

91. DESENDER, K., 1986. Investigations on Coleoptera communities in different habitats. II. The Carabid fauna from ‘Les Hautes Fagnes’ (Mont Rigi, Belgium). Bulletin de l’Institut Royal des Sciences Naturelles de Belgique d’Entomologie, 56: 15-21.

92. DESENDER, K., POLLET, M., GOOSSENS, R., 1986. The larvae of *Amara curta* Dejean, 1928 and *Amara tibialis* (Paykull, 1789) (Col., Carabidae) with notes on the life cycle of both species. Biologisch Jaarboek Dodonaea, 54: 104-115.

93. DESENDER, K., MAELFAIT, J.-P., 1986. The relation between dispersal power, commonness and biological features of carabid beetles (Col., Carabidae). Annales de la Société royale zoologique de Belgique, 116: 94.

94. DESENDER, K., POLLET, M., MAELFAIT, J.-P., 1986. Distribution of carabid beetles in Belgium: analyses on recent changes. Annales de la Société royale zoologique de Belgique, 116: 94-95.

95. DESENDER, K., MAELFAIT, J.-P., 1986. Heritability estimation and interdemic differences of wing and flight muscle development in *Pogonus chalceus* (Col., Carabidae). Annales de la Société royale zoologique de Belgique, 116: 94.

96. VAN KERCKVOORDE, M., DESENDER, K., MAELFAIT, J.-P., VANNIEUWENHUYSE, B., 1986. Composition and dispersal power of the carabid fauna in motorway verges and some urban habitats. Annales de la Société royale zoologique de Belgique, 116: 111.

97. POLLET, M., DESENDER, K., MAELFAIT, J.-P., 1986. Aspects of the feeding ecology of *Pterostichus melanarius* in a heavily grazed pasture. Annales de la Société royale zoologique de Belgique, 116: 110-111.

98. MAELFAIT, J.-P., DESENDER, K., BAERT, L., 1986. On the evolutionary origin of ecological differences between congeneric spider species. Annales de la Société royale zoologique de Belgique, 116: 102-103.

99. MAELFAIT, J.-P., DESENDER, K., 1986. Evolutionary ecology of the life cycles of some terrestrial Crustaceans. Annales de la Société royale zoologique de Belgique, 116: 100-101.

100. ALDERWEIRELDT, M., MAELFAIT, J.-P., DESENDER, K., 1986. Endangered Lycosids (Lycosidae, Araneida) of the Belgian fauna. Annales de la Société royale zoologique de Belgique, 116: 85-86.

101. MAELFAIT, J.-P., DESENDER, K., ALDERWEIRELDT, M., 1986. Life cycle and reproductive effort of Lycosid spiders (Lycosidae, Araneida). Annales de la Société royale zoologique de Belgique, 116: 101.

102. MAELFAIT, J.-P., SEYS, J., DE KEER, R., DE KIMPE, A., DESENDER, K., POLLET, M., 1986. Relations between the agricultural management and epigeic arthropod fauna of grasslands. Annales de la Société royale zoologique de Belgique, 116: 105.

103. DE KEER, R., DESENDER, K., D’HULSTER, M., MAELFAIT, J.-P., 1986. The importance of edges for the spider and beetle fauna of a pasture. Annales de la Société royale zoologique de Belgique, 116: 92-93.

104. MAELFAIT, J.-P., DESENDER, K., DE KEER, R., POLLET, M., 1986. Ecological investigations on the arthropod fauna of agro-ecosystems at Melle (Eastern Flanders, Belgium). Annales de la Société royale zoologique de Belgique, 116: 103-104.

105. POLLET, M., DESENDER, K., 1987. Carabidae van een spoorwegberm te Veldegem (West-Vlaanderen) (Coleoptera). Phegea, 15: 23-33.

106. POLLET, M., DESENDER, K., MERCKEN, L., VAN KERCKVOORDE, M., 1987. Faunistic data on Carabid beetles (Carabidae, Coleoptera) of ‘Vloetemveld’ (Zedelgem, Western Flanders). Bulletin et Annales de la Société royale belge d’Entomologie, 123: 22-28.

107. DESENDER, K., 1987. Distribution, the special case of sex-linked wing dimorphism and phenology of the life cycle in *Trichotichnus laevicollis* and *T. nitens* (Coleoptera, Carabidae). Deutsch entomologisch Zeitschrift, 34: 77-84.

108. SEGERS, H., MAELFAIT, J.-P., DESENDER, K., BAERT, L., 1987. Spider communities of woodlands with different soil and litter structure. Annales de la Société royale zoologique de Belgique, 117: 122.

109. DESENDER, K., POLLET , M., 1987. Life cycle strategies in the most abundant ground beetles from a heavily grazed pasture ecosystem. Mededelingen van de Faculteit Landbouwwetenschappen van de Rijksuniversiteit Gent, 52/2a: 191-199.

110. POLLET, M., DESENDER, K., 1987. The consequences of different life histories in ground beetles for their feeding ecology and impact on other pasture arthropods. Mededelingen van de Faculteit Landbouwwetenschappen van de Rijksuniversiteit Gent, 52/2a: 179-190.

111. DESENDER, K., BOSMANS, R., DESMET, K., 1987. Nouvelles données sur le genre *Carabus* (Coleoptera, Carabidae) d’Afrique du Nord. Annales de l’Institut national agronomique d’Algérie (El Harrach), 2(2): 46-57.

112. DESENDER, K., 1987. Ground beetles (Col., Carabidae) new or confirmed for the belgian fauna. Bulletin et Annales de la Société royale belge d’Entomologie, 123: 334-336.

113. DESENDER, K., 1987. Heritability estimates for different morphological traits related to wing development and body size in the halobiont and wing polymorphic carabid beetle *Pogonus chalceus* Marsham (Coleoptera, Carabidae). Acta Phytopathologica Entomologica Hungarica, 22: 85-101.

114. POLLET, M., DESENDER, K., 1987. Feeding ecology of grassland-inhabiting carabid beetles in relation to the availability of some prey groups. Acta Phytopathologica Entomologica Hungarica, 22: 223-246.

115. DESENDER, K., GOSSIEAUX, P., MAELFAIT, J.-P., VAN KERCKVOORDE M., POLLET, M., 1987. The position of the forest ‘Zoniënwoud’ in the distribution of woodland carabid beetles in Belgium. Acta Phytopathologica Entomologica Hungarica, 22: 329-339.

116. DESENDER, K., VAN KERCKVOORDE, M., MERTENS, J., 1987. Habitat characteristics and the composition of the carabid beetle fauna on motorway verges across a hill on sandy soil. Acta Phytopathologica Entomologica Hungarica, 22: 341-347.

117. POLLET, M., DESENDER, K., VAN KERCKVOORDE, M., 1987. Prey selection in *Loricera pilicornis* (Col., Carabidae). Acta Phytopathologica Entomologica Hungarica, 22: 425-431.

118. VAN DIJK, Th. S., ERNSTING, G., DESENDER, K., 1987. Concluding remarks of the 6th European Carabidologist’s Meeting. Acta Phytopathologica Entomologica Hungarica, 22: 455-458.

119. ERVYNCK, A., DESENDER, K., POLLET, M., 1987. Archeozoölogisch onderzoek van de beenderresten uit twee Romeinse waterputten te Burst (gem. Erpe-Mere). Archaeologia Belgica, 3: 179-182.

120. MAELFAIT, J.-P., DESENDER, K., DE KEER, R, POLLET, M., 1988. Investigations on the arthropod fauna of grasslands. In: PARK, J.R. (ed.). Environmental Management in Agriculture, European Perspectives. Belhaven Press, London, New York : 170-177.

121. DESENDER, K., 1988. Onderzoek op arthropoden in de Galapagos-archipel (Ecuador). In: Gent en haar Rijksuniversiteit in de Wereld. Uitgave Koninklijke Belgische Unie voor de Overzeese Landen en het Verenigd Europa, Gent: 19.

122. HUYSSEUNE, A., VERRAES, W., DESENDER, K., 1988. Mechanisms of branchial cartilage growth in *Astatotilapia elegans* (Teleostei: Cichlidae). Journal of Anatomy, 158: 13-30.

123. DESENDER, K., BAERT, L., MAELFAIT, J.-P., 1988. Distribution and flight activity of carabid beetles genus *Tachys* in the Galapagos archipelago. Noticias de Galàpagos, 46: 20-25.

124. DESENDER, K., 1988. Flight muscle development and dispersal in the life cycle of carabid beetles. Annales de la Société royale zoologique de Belgique, 118: 78-79.

125. POLLET, M., DESENDER, K., 1988. The occurrence of woodland inhabiting carabid beetles in relation to environmental features. Annales de la Société royale zoologique de Belgique, 118: 80.

126. DESENDER, K., ALDERWEIRELDT, M., 1988. Population dynamics of Carabid beetles and larvae in a maize field and its boundary. Zeitschrift für angewandte Entomologie, 106: 13-19.

127. DESENDER, K., 1988. The larvae of *Amara aenea* (De Geer, 1774) and *Amara familiaris* (Duftschmid, 1812) (Coleoptera, Carabidae). Bulletin et Annales de la Société royale belge d’Entomologie, 124: 153-164.

128. MAELFAIT, J.-P., BAERT, L., DESENDER, K., MERCKEN, L., 1988. Ongewervelden van onze duinen. Duinen, 2: 31-5.

129. MERCATORIS, N., DESENDER, K., DUFRENE, M., 1988. *Pterostichus rhaeticus* Heer 1837: nouvelle espèce pour le Grand-Duché de Luxembourg (Coleoptera: Carabidae). Paiperlek, Lëtzebuerger Entomologesch Zäitschrëft, 10: 20-23.

130. DESENDER, K., 1988. Spatial distribution of Carabid beetles in a pasture. Revue d’Ecologie et Biologie du Sol, 25(1): 101-113.

131. POLLET, M., MERCKEN, L., DESENDER, K., 1988. Contributions to the knowledge of Dolichopodid flies in Belgium: II. Faunistic data on the Dolichopodid fauna of some nature reserves in the Campines (Prov. Limburg, Antwerpen, Belgium) (Diptera: Dolichopodidae). Phegea, 16: 135-143.

132. DESENDER, K., 1988. Ongewervelden in de Houtsaegerduinen. Duinen, 2: 64-67, 73.

133. DESENDER, K., POLLET, M., 1988. Sampling pasture carabids with pitfalls: evaluation of species richness and precision. Mededelingen van de Faculteit Landbouwwetenschappen van de Rijksuniversiteit Gent, 53/3a: 1109-1117.

134. POLLET, M., DESENDER, K., 1988. Quantification of prey uptake in pasture inhabiting carabid beetles. Mededelingen van de Faculteit Landbouwwetenschappen van de Rijksuniversiteit Gent, 53/3a: 1119-1129.

135. POLLET, M., MEUFFELS, H., MERCKEN, L., DESENDER, K., 1988. Faunistic data on the Dolichopodid fauna (Dolichopodidae, Diptera) of some habitats in the Famenne (Prov. Namur, Belgium). Biologisch Jaarboek Dodonaea, 56: 50-61.

136. MAELFAIT, J.-P., DESENDER, K., DE KEER, K., 1988. The arthropod community of the edge of an intensively grazed pasture. In : SCHREIBER, K.-F. (ed.). Connectivity in Landscape Ecology. Münstersche Geographische Arbeiten 29, Munster : 115-117.

137. POLLET, M., MERCKEN, L., DESENDER, K., 1989. A note on the detailed distribution and diel activity of riparian dolichopodid species (Dolichopodidae, Diptera). Bulletin et Annales de la Société royale belge d’Entomologie, 124: 248-253.

138. POLLET, M., DESENDER, K., 1989. Boekbespreking : ‘De Loopkevers (Cicindelidae en Carabidae) van Nederland (BOEKEN, M.). Jeugdbondsuitgeverij, Utrecht 1987, 155 pp. Phegea, 16: 79-80.

139. DESENDER, K., TURIN, H., 1989. Loss of habitats and changes in the composition of the ground- and tiger beetle fauna in four West-European countries since 1950 (Coleoptera: Carabidae, Cicindelidae). Biological Conservation, 48: 277-294.

140. DESENDER, K., 1989. Heritability of wing development and body size in a Carabid beetle, *Pogonus chalceus* Marsham, and its evolutionary significance. Oecologia (Berlin), 78: 513-520.

141. DE KEER, R., ALDERWEIRELDT, M., DECLEER, K., SEGERS, H., DESENDER, K., MAELFAIT, J.-P., 1989. Horizontal distribution of the spider fauna of intensively grazed pastures under the influence of diurnal activity and grass height. Zeitschrift für angewandte Entomologie, 107: 455-473.

142. DESENDER, K., BUNGENEERS, J., ERVYNCK, A., 1989. Keverresten uit de waterput. Scharnier, 6: 5.

143. ERVYNCK, A., DESENDER, K., 1989. Dierenresten uit drie Romeinse waterputten te Burst. De Merenaar, 1989, 1-7.

144. BAERT, L., MAELFAIT, J.-P., DESENDER, K., 1989. Spider communities of Isla Santa Cruz (Galápagos Archipelago-Ecuador). Reports from the Department of Biology, University of Turku, Finland, 19: 4.

145. MAELFAIT, J.-P., ALDERWEIRELDT, M., DESENDER, K., BAERT, L., 1989. Lycosid Spiders of the Belgian Coastal Dunes. Reports from the Department of Biology, University of Turku, Finland, 19: 61.

146. MAELFAIT, J.-P., JOCQUE, R., BAERT, L., DESENDER, K., 1989. Heathland Management ans Spider communities. Reports from the Department of Biology, University of Turku, Finland, 19: 62.

147. ALDERWEIRELDT, M., DESENDER, K., HUBLE, J., POLLET, M., 1989. Een ecologische analyse van de spinnen- en loopkeverfauna van vijf boshabitaten in de Famenne. Natuurwetenschappelijk Tijdschrift, 71: 8-17.

148. BAERT, L., MAELFAIT, J.-P., DESENDER, K., 1989. Results of the Belgian 1986-expedition: Araneae and provisional checklist of the spiders of the Galapagos archipelago. Bulletin de l’Institut Royal des Sciences Naturelles de Belgique, Entomologie, 58: 29-54.

149. DESENDER, K., BAERT, L., MAELFAIT, J.-P., 1989. Contribution to the knowledge of the Carabid beetles of Galàpagos (Ecuador). Bulletin de l’Institut Royal des Sciences Naturelles de Belgique, Entomologie, 58: 55-65.

150. DESENDER, K., 1989. Dispersievermogen en Ecologie van Loopkevers (Coleoptera, Carabidae) in België: een evolutionaire benadering. Studiedocument Nr 54, Koninklijk Belgisch Instituut voor Natuurwetenschappen, Brussel, 135 pp.

151. ALDERWEIRELDT, M., DESENDER, K., 1989. Faunistisch araneologisch onderzoek van intensief bewerkte akkers en hun randen in België: een korte evaluatie. Phegea, 17: 161-164.

152. DESENDER, K., ALDERWEIRElDT, M., POLLET, M., 1989. Field edges and their importance for polyphagous predatory arthropods. Mededelingen van de Faculteit Landbouwwetenschappen van de Rijksuniversiteit Gent, 54/3a: 823-833.

153. POLLET, M., DESENDER, K., 1989. Prey uptake in subdominant, small to medium-sized carabid beetles from a pasture ecosystem. Mededelingen van de FaculteitLandbouwwetenschappen van de Rijksuniversiteit Gent, 54/3a: 809-822.

154. DECLEER, K., ALDERWEIRELDT, M., SEGERS, H. , HUBLE, J., DESENDER, K., 1989. Inventarisatie van de spinnen (Araneae) in het natuureducatief reservaat “Jalna” en een nabijgelegen weidebiotoop te Heure-en-Famenne (Prov. Namen). Nieuwsbrief van de Belgische Arachnologische Vereniging, 12: 15-22.

155. DESENDER, K., 1989. Ecomorphological adaptations of riparian carabid beetles. Verhandelingen van het Symposium “Invertebraten van België”, Brussel 25-26 nov. 1988, pp. 309-314.

156. GASPAR, Ch., DUFRENE, M., THIRION, C., FAGOT, J., DESENDER, K., MAELFAIT, J.-P., RASMONT, P., DESIERE, M., 1989. Recherches sur l’écosytème forêt, Biocénose des Coléoptères. Verhandelingen van het symposium «Invertebraten van België», Brussel 25-26 nov. 1988, p. 293-300.

157. MAELFAIT, J.-P., DESENDER, K., BAERT, L., 1989. Some examples of the practical use of spiders and carabid beetles as ecological indicators. Verhandelingen van het Symposium “Invertebraten van België”, Brussel 25-26 nov. 1988, p. 437-42.

158. POLLET, M., DESENDER, K., ALDERWEIRELDT, M., 1989. Evaluation of the seasonality of predation and the degree of food specialization in grassland-inhabiting carabid beetles (Col., Carabidae). Verhandelingen van het Symposium “Invertebraten van België, Brussel 25-26 nov. 1988, pp. 331-338.

159. MAES, D., DECLEER, K., DESENDER, K., VERHAEGHE, B., 1989. De loopkeverfauna (Coleoptera: Carabidae) van het natuurreservaat de Blankaart (Woumen, West-Vlaanderen). Bulletin et Annales de la Société royale belge d’Entomologie, 125: 309-319.

160. MAELFAIT, J.-P., ALDERWEIRELDT, M., DESENDER, K., BAERT, L., 1989. Lycosid spiders of the Belgian coast. Bulletin et Annales de la Société royale belge d’Entomologie, 125: 327-332.

161. DESENDER, K., DE DIJN, B., 1989. The *Calosoma* species of the Galapagos archipelago. I. Redescription and distribution of the species. Bulletin de l’Institut Royal des Sciences Naturelles de Belgique, Entomologie, 59: 131-144.

162. BAERT, L., MAELFAIT, J.-P., DESENDER, K., 1989. Results of the Belgian 1988-expedition to the Galapagos islands: Araneae. Bulletin de l’Institut Royal des Sciences Naturelles de Belgique, Entomologie, 59: 5-22.

163. DESENDER, K., ERVYNCK, A., 1990. In: Het archeologisch onderzoek van het Eindhovense heuvelterrein. Loopkevers (Arts, N., ed.), pp. 165-173. Eindhoven.

164. ALDERWEIRELDT, M., DESENDER, K., 1990. Microhabitat preference of spiders (Araenae) and carabid beetles (Coleoptera, Carabidae) in maize fields. Mededelingen van de Faculteit Landbouwwetenschappen van de Rijksuniversiteit Gent, 55 (2b): 501-510.

165. POLLET, M., DESENDER, K., 1990. Investigating the food passage in *Pterostichus melanarius* (Coleoptera, Carabidae): an attempt to explain its feeding behaviour. Mededelingen van de Landbouwwetenschappen van de Rijksuniversiteit Gent, 55 (2b): 527-540.

166. DESENDER, K., BAERT, L., MAELFAIT, J.-P., 1990. Distribution and speciation of carabid beetles in Galapagos. Belgian Journal of Zoology, 120 (Suppl. 1): 24.

167. DESENDER, K., MAELFAIT, J.-P., BAERT, L., 1990. Carabid beetles as bio-indicators in Belgian coastal dunes. Belgian Journal of Zoology, 120 (Suppl. 1): 25.

168. MAELFAIT, J.-P., BAERT, L., DESENDER, K., 1990. Faunal interests of wet dune grasslands along the belgian coast. Belgian Journal of Zoology, 120 (Suppl. 1): 46.

169. MAELFAIT, J.-P., BAERT, L., DESENDER, K., 1990. The spider fauna of a north and south facing motorway verge. Belgian Journal of Zoology, 120 (Suppl. 1): 45.

170. MAELFAIT, J.-P., JOCQUE, R., BAERT, L., DESENDER, K., 1990. Effects of different management practices on the spider communities of dry heathland. Belgian Journal of Zoology, 120 (Suppl. 1): 45.

171. MAELFAIT, J.-P., SEGERS, H., BAERT, L., DESENDER, K., 1990. The relation between the heterogeneity of forest complexes and the richness of their spider fauna. Belgian Journal of Zoology, 120 (Suppl. 1): 46.

172. DESENDER, K., BAERT, L., MAELFAIT, J.-P., 1990. Carabid beetles of Galapagos (Ecuador) collected during the Belgian 1988-expedition. Bulletin van het Koninklijk Belgisch Instituut voor Natuurwetenschappen, Entomologie, 60: 49-54.

173. DESENDER, K., BAERT, L., MAELFAIT, J.-P., 1990. Evolutionary Ecology of Carabids in the Galapagos Archipelago. In: The role of Ground Beetles in Ecological and Environmental Studies (Stork, N.E., ed.) : 13-20.

174. MAELFAIT, J.-P., DESENDER, K., 1990. Possibilities of Short-term Carabid Sampling for Site Assessment Studies. In: The role of Ground Beetles in Ecological and Environmental Studies (Stork, N.E., ed.) : 217225.

175. MAELFAIT, J.-P., DESENDER, K., BAERT, L., 1990. Carabids as Ecological Indicators for Dune Management Evaluation. In: The role of Ground Beetles in Ecological and Environmental Studies (Stork, N.E., ed.) : 331333.

176. ALDERWEIRELDT, M., DESENDER, K., 1990. Variation of Carabid Diel Activity Patterns in Pastures and Cultivated Fields. In: The role of Ground Beetles in Ecological and Environmental Studies (Stork, N.E., ed.) : 335338.

177. DUFRENE, M., BAGUETTE, M., DESENDER, K., MAELFAIT, J.-P., 1990. Evaluation of Carabids as Bioindicators : a Case study in Belgium. In: The Role of Ground Beetles in Ecological and Environmental Studies (Stork, N.E., ed.) : 377-381.

178. DESENDER, K., M. ALDERWEIRELDT, 1990. The carabid fauna of maize fields under different rotation regimes. Mededelingen van de Faculteit Landbouwwetenschappen van de Rijksuniversiteit Gent, 55 (2b): 493-500.

179. DESENDER, K.,, DE DIJN, B., 1990. The *Calosoma* species (Coleoptera, Carabidae) of the Galápagos archipelago. II. Discriminant analyses and species identification key. Bulletin de l’Institut Royal des Sciences Naturelles de Belgique, Entomologie, 60: 55-68.

180. DESENDER, K., 1990. Preliminary note on *Asaphidion curtum* (Heyden, 1870) and *A. stierlini* (Heyden, 1880) : two Carabid beetles new for the belgian fauna (Coleoptera : Carabidae). Bulletin et Annales de la Société royale belge d’Entomologie, 126: 179-180.

181. DESENDER, K., 1990. Les Coléoptères Carabiques d’un site remarquable à Corphalie et leur valeur pour la conservation de la nature en Belgique. Bulletin et Annales de la Société royale belge d’Entomologie, 126: 213-216.

182. DESENDER, K., BAERT, L., HUYSSEUNE, A., 1990. Ground and Tiger beetles (Coleoptera, Carabidae) of the «Gaume» region in Belgium. Bulletin et Annales de la Société royale belge d’Entomologie, 126: 186-194.

183. BAERT, L., DESENDER, K., MAELFAIT, J.-P., 1990. A preliminary study of the spider communities of Isla Isabela (Galapagos archipelago, Ecuador). Comptes rendus du XIIème Colloque européen d’Arachnologie. Bulletin de la Société européenne d’Arachnologie, N° hors série 1: 10-16.

184. BAERT, L., DESENDER, K., MAELFAIT, J.-P., 1990. Arachnological and Carabidological Investigations in Galápagos During February and March 1986 : A Preliminary Report. Annual Report 1986-1987 of the Charles Darwin Research Station, Galápagos, Ecuador : 55-57.

185. BAERT, L., DESENDER, K., MAELFAIT, J.-P., 1990. Estudio Aracnologico y Carabidologico en Galápagos durante Febrero y Marzo de 1986 : un Informe Preliminar. Informe Anual 1986-1987 de la Estacíon Cientifica Charles Darwin, Galápagos, Ecuador : 58-60.

186. DESENDER, K., ALDERWEIRELDT, M., 1990. Yearly and seasonal variation of carabid diel activity in pastures and cultivated fields. Revue d’Ecologie et de Biologie du Sol, 27(4): 423-433.

187. MAELFAIT, J.-P., JOCQUE, R., BAERT, L., DESENDER, K., 1990. Heathland management and spiders. Acta Zoologica Fennica, 190: 261-266.

188. DESENDER, K., HUYSSEUNE, A., MAELFAIT, J.-P., 1991. Enkele gegevens over de loopkeverfauna van ‘De Groenendijk’ (‘O.L.V. Ter Duinen’, Oostduinkerke) en ‘De Schuddebeurze’ en omgeving (Westende). Duinen, 5: 57-63.

189. MAELFAIT, J.-P., DESENDER, K., BAERT, L., POLLET, M., 1991. Duinbeheer en ongewervelden. 1. Achtergronden en algemeen opzet van het onderzoek. Duinen, 5: 18-26.

190. DESENDER, K., ERVYNCK, A., 1991. Archeologie op zes poten. Archaeologia Mediaevalis, 14 : 9-10.

191. DESENDER, K.,, MAELFAIT, J-P., 1991. Carabid beetles from ‘De Zandpanne’, a small dune nature reserve along the belgian coast. Bulletin et Annales de la Société royale belge d’Entomologie, 127: 15-18.

192. BAERT, J., DESENDER, K., MAELFAIT, J.-P., 1991. Spider communities of Isla Santa Cruz (Galápagos, Ecuador). Journal of Biogeography, 18 : 333-340.

193. BAERT, L., DESENDER, K., HUYSSEUNE, A., 1991. Spinnen van Belgisch Lotharingen (Provincie Luxemburg, België). Nieuwsbrief Belgische Arachnologische Vereniging, 6(2): 1-5.

194. ALDERWEIRELDT, M., DESENDER, K., MAELFAIT, J.-P., POLLET, M., 1991. Faunistische bijdrage tot de kennis van de araneofauna van enkele weinig onderzochte regio’s in België. Deel. 1. De Westhoek en het Zuidwestvlaamse heuvelland. Nieuwsbrief Belgische Arachnologische Vereniging, 6(3): 25.

195. ALDERWEIRELDT, M., MAELFAIT, J.-P., DESENDER, K., POLLET, M., 1991. Faunistische bijdrage tot de kennis van de araneofauna van enkele weinig onderzochte regio’s in België. Deel. 2. Tielt, Waregem en het Noorden van Henegouwen. Nieuwsbrief Belgische Arachnologische Vereniging, 6(3): 68.

196. DESENDER, K., BAERT, L., MAELFAIT, J.-P., 1991. Evolutionary systematics of *Calosoma* Weber carabid beetles of the Galápagos archipelago (Coleoptera, Carabidae). In: Advances in Coleopterology (ZUNINO, M., BELLES, X., BLAS, M., eds.): 193-200.

197. ALDERWEIRELDT, M., POLLET, M., DESENDER, K., 1991. Abundance and dynamics of adult and larval Coleoptera in different agro-ecosystems. In: Advances in Coleopterology (ZUNINO, M., BELLES, X., BLAS, M., eds.): 223-231.

198. ERVYNCK, A., DESENDER, K., POLLET, M., 1991. Organische resten uit de waterput D te Burst (gem. ErpeMere). Archeologie in Vlaanderen, 1: 129133.

199. DESENDER, K., MAELFAIT, J.-P., BAERT, L., 1991. Carabid beetles as ecological indicators in dune management. Elytron, Supplement 5(1): 239-247.

200. DESENDER, K., 1991. Etude des restes d’insectes et principalement des Coléoptères (Coleoptera, Carabidae) provenant du site archéologique des Jardins du Carrousel (Paris). Les Jardins du Carrousel à Paris. Fouilles 1989-1990. III. Les rapports des spécialistes (ed. P. Van Ossel), p. 143, Paris

201. BAERT, L., DESENDER, K., PECK, S., 1992. New data on the Neuroptera of the Galápagos Islands, Ecuador. Bulletin van het Koninklijk Belgisch Instituut voor Natuurwetenschappen, Entomologie, 62: 143-147.

202. DESENDER, K., BAERT, L., MAELFAIT, J.-P., 1992. Distribution and speciation of carabid beetles in the Galápagos Archipelago (Ecuador). Bulletin van het Koninklijk Belgisch Instituut voor Natuurwetenschappen, Entomologie, 62: 57-66.

203. DESENDER, K., BAERT, L., MAELFAIT, J.-P., 1992. El Niño-events and the establishment of ground beetles in the Galápagos Archipelago. Bulletin van het Koninklijk Belgisch Instituut voor Natuurwetenschappen, Entomologie, 62: 67-74.

204. ALDERWEIRELDT, M., DESENDER, K., 1992. Diel activity patterns of carabid beetles in some crop-rotated fields studied by means of time-sorting pitfall traps. Mededelingen van de Faculteit Landbouwwetenschappen van de Rijksuniversiteit Gent, 57/3a: 603-612.

205. ALDERWEIRELDT, M., DESENDER, K., 1992. Faunistische bijdrage tot de kennis van de araneofauna van enkele weinig onderzochte regio’s in België. Deel. 3. Het zuiden van Oost-Vlaanderen. Nieuwsbrief Belgische Arachnologische Vereniging, 7(2): 3-6.

206. DESENDER, K., BAGUETTE, M., DUFRENE, M., MAELFAIT, J.-P., 1992. A state of knowledge on the distribution of carabids in Belgium and northern France. In : Van Goethem, J.L., Grootaert, P. (eds.). Faunal inventories of sites for cartography and nature conservation. Proceedings of the 8th International Colloquium of the European Invertebrate Survey, Brussels, 09-10 September 1991, pp 67-73.

207. MAELFAIT, J.-P., COULON, G., DESENDER, K., 1992. The Flemish legislation on the protection of Coleoptera in European perspective. In : Van Goethem, J.L., Grootaert, P. (eds.). Faunal inventories of sites for cartography and nature conservation. Proceedings of the 8th International Colloquium of the European Invertebrate Survey, Brussels, 09-10 September 1991, pp 81-90.

208. DESENDER, K., BAERT, L., 1992. De loopkeverfauna van het Militair Domein te Lombardsijde (Coleoptera, Carabidae). Bulletin et Annales de la Société royale belge d’Entomologie, 128: 263-266.

209. DESENDER, K., 1992. Amara majuscula, a carabid beetle new for the Belgian fauna: overlooked or only a temporary visitor? Bulletin et Annales de la Société royale belge d’Entomologie, 128: 298-301.

210. DESENDER, K., ALDERWEIRELDT, M., MAELFAIT, J-P., 1992. Dry heathland vegetation structure and carabid beetles. Proceedings of the 4th ECE/XIII.SIEEC, Gödöllö 1991, Vol. 1: 149-152.

211. DESENDER, K., MAELFAIT, J.-P., BAERT, L., 1992. Monitoring carabid beetles in Belgian coastal dunes. Proceedings of the 4th ECE/XIII.SIEEC, Gödöllö 1991, Vol. 1: 153-158.

212. MAELFAIT, J.-P., DESENDER, K., POLLET, M., SEGERS, H., BAERT, L., 1992. Carabid beetle and spider communities of Belgian forest stands. Proceedings of the 4th ECE/XIII.SIEEC, Gödöllö 1991, Vol. 1: 187-194.

213. LENTACKER, A., BAKELS, C.C., VERBEECK, M., DESENDER, K., 1992. The archaeology, fauna and flora of a Roman well at Erps-Kwerps (Brabant, Belgium). Helinium, 32(1-2) :110-131.

214. BAERT, L., DESENDER, K., 1993. De spinnenfauna van het Militair Domein te Lombardsijde (Araneae). Nieuwsbrief Belgische Arachnologische Vereniging, 8(1): 15-20.

215. DESENDER, K., 1993. Habitatpreferentie en levenscyclus van loopkevers (Coleoptera, Carabidae) in het duinengebied ‘Domein Theunis’ (Oostduinkerke). Duinen, 7(4):138-147.

216. LENTACKER, A., VAN NEER, W., DESENDER, K., 1993. Achézoologie. In: Braives Gallo-Romain. V. La fortification du bas-empire (Hackens, T., editeur). Publications d’histoire de l’art et d’archéologie de l’UCL, 83, Louvain-la-Neuve, pp. 284-339.

217. DESENDER, K., 1993. Carabid beetles (Coleoptera, Carabidae) from hedgerows in the grand Duchy of Luxemburg: species inventory, additions to the fauna and data on species with special biogeographic interest. Travaux scientifiques du Musée national d’histoire naturelle de Luxembourg, 20: 155-164.

218. DESENDER, K., DUFRENE, M., LOREAU, M., LUFF, M.L., MAELFAIT, J.-P. (editors), 1994. Carabid beetles: ecology and evolution. Kluwer Academic Publishers, Dordrecht, Boston, London, 474 pp.

219. DESENDER, K., DUFRENE, M., LOREAU, M., LUFF, M.L., MAELFAIT, J.-P., 1994. Introduction. In: Carabid beetles: ecology and evolution (Desender, K. et al., eds.). Kluwer Academic Publishers, Dordrecht, Boston, London, pp. xi-xii.

220. DESENDER, K., DUFRENE, M., MAELFAIT, J.-P., 1994. Long term dynamics of carabid beetles in Belgium: a preliminary analysis on the influence of changing climate and land use by means of database covering more than a century. In: Carabid beetles: ecology and evolution (Desender, K. et al., eds.). Kluwer Academic Publishers, Dordrecht, Boston, London, pp. 247-252.

221. ERVYNCK, A., DESENDER, K., PIETERS, M., BUNGENEERS, J., 1994. Carabid beetles as palaeo-ecological indicators in archaeology. In: Carabid beetles: ecology and evolution (Desender, K. et al., eds.). Kluwer Academic Publishers, Dordrecht, Boston, London, pp. 261-266.

222. MAELFAIT, J.-P., DESENDER, K., DUFRENE, M., 1994. Carabid beetles and nature conservation research in Belgium: a review. In: Carabid beetles: ecology and evolution (Desender, K. et al., eds.). Kluwer Academic Publishers, Dordrecht, Boston, London, pp. 319-323.

223. ALDERWEIRELDT, M., DESENDER, K., 1994. Belgian carabidological research on high-input agriculural fields and pastures: a review. In: Carabid beetles: ecology and evolution (Desender, K. et al., eds.). Kluwer Academic Publishers, Dordrecht, Boston, London, pp. 409-415.

224. DE MEESTER, L., VANDENBERGHE, J., DESENDER, K., DUMONT, H.J., 1994. Genotype-dependent daytime vertical distribution of *Daphnia magna* in a shallow pond. Belgian Journal of Zoology, 124: 3-9.

225. DESENDER, K., ERVYNCK, A., 1994. Onderzoek van loopkeverresten: Karolingische waterput, Zerkegem (W.-Vl.) en Hoge Dijken, Roksem (W.-Vl.). Archaeologia Mediaevalis, 17 : 9.

226. MAELFAIT, J.-P., DESENDER, K., BAERT, L., 1994. Ecological diversity and population dynamics of ground beetles and spiders of coastal dunes: research with applications for nature conservation. In: Biodiversity: study, exploration, conservation (Hoffmann, M., Van Der Veken, P., eds.). Proceedings of a Symposium organized by the Royal Society of Natural Sciences Dodonaea, Univ. Gent, 18 Nov. 1992: 168-169.

227. BAERT, L., MAELFAIT, J.-P., DESENDER, K., 1994. Evolutionary ecology, systematics and population genetics of terrestrial arthropods in the Galápagos Archipelago (Ecuador). In: Biodiversity: study, exploration, conservation (Hoffmann, M., Van Der Veken, P., eds.). Proceedings of a Symposium organized by the Royal Society of Natural Sciences Dodonaea, Univ. Gent, 18 Nov. 1992: 170-171.

228. DESENDER, K., BAERT, L., MAELFAIT, J.-P., 1994. Diversity in distribution patterns and speciation mechanisms of carabid beetles in the Galápagos Archipelago (Ecuador). In: Biodiversity: study, exploration, conservation (Hoffmann, M., Van Der Veken, P., eds.). Proceedings of a Symposium organized by the Royal Society of Natural Sciences Dodonaea, Univ. Gent, 18 Nov. 1992: 172-173.

229. DESENDER, K., MAELFAIT, J.-P., STEVENS, J., ALLEMEERSCH, L., 1994. Loopkevers langs de Grensmaas. LIKONA, Jaarboek 1993: 41-50.

230. DESENDER, K., ERVYNCK, A., 1994. Wat ruist daar door het struikgewas. De middeleeuwse loopkevers van het Heuvelterrein. In: Arts, N. (ed.). Sporen onder de Kempische stad. Archeologie, ecologie en vroegste geschiedenis van Eindhoven. Museum Kempenland Eindhoven: 295-301.

231. JANSSEN, M., MAELFAIT, J.-P., ALDERWEIRELDT, M., DESENDER, K., 1994. Faunistische bijdrage tot de kennis van de araneofauna van enkele weinig onderzochte regio’s in België. Deel 4. Noord-Limburg. Nieuwsbrief Belgische Arachnologische Vereniging, 9(3): 72-81.

232. HOLLEVOET, Y., COOREMANS, B., DESENDER, K., ERVYNCK, A., 1995. Een Karolingische vlechtwaterput uit Zerkegem (gem. Jabbeke, prov. West-Vlanderen): culturele en ecologische archaeologica. Archeologie in Vlaanderen III - 1993: 243-254.

233. DESENDER, K., 1995. De loopkeverfauna van het domein ‘Jalna’ en zijn onmiddellijke omgeving: een kort overzicht. Determinatielijsten Flora en Fauna, Domein Jalna, 4pp.

234. DESENDER, K., 1995. Carabidae. In: Enumeratio Coleopterorum Belgicae 1 (Coulon, G., ed.), Brussel, Koninklijke Belgische Vereniging voor Entomologie, pp. 13-28.

235. DESENDER, K., D. MAES, 1995. Carabid beetles new to or confirmed for the Belgian fauna. Bulletin et Annales de la Société royale belge d’Entomologie, 131:213-223.

236. DESENDER, K., MAES, D., MAELFAIT, J.-P., M. VAN KERCKVOORDE, M., 1995. Een gedocumenteerde Rode lijst van de zandloopkevers en loopkevers van Vlaanderen. Mededelingen van het Instituut voor Natuurbehoud 1995 (1):1-208.

237. BAERT, L., MAELFAIT, J.-P., DESENDER, K., 1995. Distribution of the arachnid species of the Orders Scorpiones, Solifugae, Amblypygi, Schizomida, Opiliones and Pseudoscorpiones in Galápagos. Bulletin van het Koninklijk Belgisch Instituut voor Natuurwetenschappen, Entomologie, 65: 5-19.

238. DESENDER, K., BAERT, L., 1995. Carabid beetles as bio-indicators in Belgian coastal dunes: a long term monitoring project. Bulletin van het Koninklijk Belgisch Instituut voor Natuurwetenschappen, Entomologie, 65: 35-54.

239. MAELFAIT, J.-P., DESENDER, K., 1995. Field margins as refuges and potential corridors for arthropods in the Flemish agricultural landscape. Field Margins Newsletter, 5: 7-9.

240. DESENDER, K., 1996. Diversity and dynamics of coastal dune carabids. Annales Zoologici Fennici, 33 (1): 65-76.

241. BAERT, L., DESENDER, K., MAELFAIT, J.-P., 1996. Arachnological and carabidological study in Galápagos during February and March 1988. Charles Darwin Research Station, 1988-1989 Annual Report: 76-78.

242. DESENDER, K., L. BAERT, 1996. Easter Island revisited: carabid beetles. The Coleopterists Bulletin, 50 (4):343-356.

243. DESENDER, K., L. BAERT, 1996. The Coleoptera of Easter Island. Bulletin van het Koninklijk Belgisch Instituut voor Natuurwetenschappen, Entomologie, 66: 27-50.

244. ERVYCNK, A., DEMIDDELE, H., DESENDER, K., J. SCHELVIS, 1996. Loopkevers, mijten en kiezelwieren: bewijsmateriaal bij archaeologische milieureconstructies. Tijdschrift voor Ecologische Geschiedenis, 1: 9-16.

245. MAELFAIT, J.-P., BAERT, L., DESENDER, K., 1997. Effects of groundwater catchment and grassland management on the spider fauna of the dune nature reserve ‘De Westhoek’ (Belgium). Proceedings of the 16th European Colloquium on Arachnology, Siedlce, 10/3/1977: 221-235.

246. DESENDER, K., 1997. Book review: Arthropods natural enemies in arable land II. Survival, reproduction and enhancement. Entomologia Experimentalis et Applicata, 84: 105-107.

247. DESENDER, K., BAERT, L., 1997. Conservation of terrestrial arthropods on Easter Island as exemplified by the beetle fauna. Conservation Biology, 11: 836-838.

248. BAERT, L., LEHTINEN, P., DESENDER, K., 1997. The spiders (Araneae) of Rapa Nui (Easter Island). Bulletin van het Koninklijk Belgisch Instituut voor Natuurwetenschappen, Entomologie, 67: 9-32.

249. DESENDER, K., BACKELJAU, TH., DELAHAYE, K., DE MEESTER, L., 1998. Age and size of European saltmarshes and the population genetic consequences for ground beetles. Oecologia, 114:503-513.

250. BAUER, Th., DESENDER, K., MORWINSKY, Th., BETZ, O., 1998. Eye morphology reflects habitat demands in three closely related ground beetle species (Coleoptera: Carabidae). Journal of Zoology, London, 245:467-472.

251. DESENDER, K., VANDEN BUSSCHE, C. , 1998. Ecological diversity, assemblage structure and life cycles of ground beetles (Col., Carabidae) in the forest of Ename (Eastern Flanders, Belgium). Bulletin van het Koninklijk Belgisch Instituut voor Natuurwetenschappen, Entomologie, 68: 37-52.

252. DE MEESTER, L., MICHIELS, E., VANOVERBEKE, J., COUSYN, C., DEGANS, H., AUDENAERT, E., DESENDER, K., MAELFAIT, J.-P., 1998. Population genetic studies on zooplankton: patterns of genetic variation in organisms inhabiting insular habitats as a means of comparing the merits of different nature conservation strategies. In: Populations: natural and manipulated (Beeckman, T., Camelbeke, K., eds.). Proceedings of a Symposium organized by the Royal Society of Natural Sciences Dodonaea, Univ. Gent, 29 Oct. 1997: 129-131.

253. DESENDER, K., BACKELJAU, TH., DELAHAYE, K., DE MEESTER, L., 1998. Age and size of European saltmarshes and the population genetic consequences: a case study on two ground beetle species. In: Populations: natural and manipulated (Beeckman, T., Camelbeke, K., eds.). Proceedings of a Symposium organized by the Royal Society of Natural Sciences Dodonaea, Univ. Gent, 29 Oct. 1997: 142.

254. DESENDER, K., VERDYCK, P., BAERT, L., VERHEYEN, E., 1998. Population genetic structure and differentiation in the Galápagos caterpillar hunter *Calosoma granatense* (Col., Carabidae). In: Populations: natural and manipulated (Beeckman, T., Camelbeke, K., eds.). Proceedings of a Symposium organized by the Royal Society of Natural Sciences Dodonaea, Univ. Gent, 29 Oct. 1997: 143-144.

255. VERDYCK, P., DESENDER, K., BAERT, L., VERHEYEN, E., 1998. Molecular phylogeography of the phytophagous Galápagos beetle *Nesaecrepida darwini* Mutchler (Coleoptera : Chrysomelidae). In: Populations: natural and manipulated (Beeckman, T., Camelbeke, K., eds.). Proceedings of a Symposium organized by the Royal Society of Natural Sciences Dodonaea, Univ. Gent, 29 Oct. 1997: 199-200.

256. VERDYCK, P., DESENDER, K., 1998. Population genetics of the endemic flea beetle *Nesaecrepida darwini* on the Galápagos Archipelago. In: Populations: natural and manipulated (Beeckman, T., Camelbeke, K., eds.). Proceedings of a Symposium organized by the Royal Society of Natural Sciences Dodonaea, Univ. Gent, 29 Oct. 1997: 201-202.

257. DESENDER, K. , BOSMANS, R., 1998. Ground beetles (Coleoptera, Carabidae) on set-aside fields in the Campine region and their importance for nature conservation in Flanders (Belgium). Biodiversity and Conservation, 7: 1485-1493.

257b.HENDRICKX, F, MAELFAIT, J-P, DESENDER, K **(**1998) - Arthropodes terrestres le long de l’Escaut et de la Meuse. Notes fauniques de Gembloux, 35: 70-82.

258. DESENDER, K., MAELFAIT, J.-P., 1999. Diversity and conservation of terrestrial arthropods in tidal marshes along the River Schelde: a gradient analysis. Biological Conservation, 87:221-229.

259. DESENDER, K., MAELFAIT, J.-P., BAERT, L., VERDYCK, P., 1999. Conservation on Volcán Alcedo (Galápagos): terrestrial invertebrates and the impact of introduced feral goats. Biological Conservation, 87:303-310.

260. DESENDER, K., SERRANO, J, 1999. A genetic comparison of Atlantic and Mediterranean populations of a saltmarsh beetle. Belgian Journal of Zoology., 129: 83-94.

261. VERDYCK, P., DESENDER, K. 1999. Hierarchical population genetic analysis reveals metapopulation structure in a phytophagous Galápagos beetle. Belgian Journal of Zoology, 129: 95-104.

262. DESENDER, K., ERVYNCK, A.,, TACK, G., 1999. Beetle diversity and historical ecology of woodlands in Flanders. Belgian Journal of Zoology, 129: 139-156.

263. ERVYNCK, A., DESENDER, K., VAN NEER, W., 1999. Archeozoölogisch onderzoek van middeleeuwse vindplaatsen op het IAP. Archaeologia Mediaevalis, 22: 5-6.

264. WOUTERS, W., COOREMANS, B., DESENDER, K., ERVYNCK, A., VAN STRYDONCK, M., 1999. Archeologisch en ecologisch onderzoek van een vroegmiddeleeuwse waterput te Kasterlee (prov. Antwerpen). Archeologie in Vlaanderen V 1995/1996: 97-109.

265. PIETERS, M., BOUCHET, F., COOREMANS, B., DESENDER, K., ERVYNCK, A.,, VAN NEER, W., 1999. Granaatappels, een zeeëngel en rugstreeppadden. Een greep uit de inhoud van een bakstenen beerput uit het 15de-eeuwse Raversijde (Oostende, prov. West-Vlaanderen). Resten van loopkevers. Archeologie in Vlaanderen V 1995/1996: 193-224.

266. MAES, D., DESENDER, K.,, ALDERWEIRELDT, M., 1999. Bijzondere loopkevers en spinnen in vier biotooptypen in Heppen-Leopoldsburg. LIKONA, Jaarboek 1998: 63-71.

267. DESENDER, K., MAELFAIT, J.-P.,, MAES, D., 1999. Loopkevers. Natuurrapport 1999. Toestand van de natuur in Vlaanderen: cijfers voor het beleid. Instituut voor Natuurbehoud: 78-80.

268. DE WOLF, H., VAN GOETHEM, J.L., DESENDER, K., MEDEIROS, R., VAN RIEL, P., BACKELJAU, T. 1998. ITS1 sequence variation in *Ovatella myosotis* Draparnaud, 1801 (Mollusca: Basommatophora). - Abstracts of the World Congress of Malacology, Washington, 25-30 July 1998, p. 87.

269. VERDYCK, P., DESENDER, K., 1999. Loopkevers (Coleoptera Carabidae) van twee bossen in de gemeentes Schoten en Brasschaat: het natuurreservaat ’t Asbroek (domein Amerlo) en het Peerdsbos. Bulletin de la Société royale belge d’Entomologie, 135: 179-180.

270. NIEMELÄ, J., KOTZE, J., ASHWORTH, A., BRANDMAYR, P., DESENDER, K., NEW, T., PENEV, L., SAMWAYS, M.,, SPENCE, J., 1999. The search for common anthropogenic impacts on biodiversity: a global network. Journal of Insect Conservation, 3: 1-7.

271. THIJS, N., DESENDER, K., 2000. Loopkevers en zweefvliegen in de Winterbeekvallei. LIKONA, Jaarboek 1998: 39-47.

272. DESENDER, K., 2000. Flight muscle development and dispersal in the life cycle of carabid beetles: patterns and processes. Bulletin van het Koninklijk Belgisch Instituut voor Natuurwetenschappen, Entomologie, 70: 13-31.

273. VERDYCK, P., DESENDER, K., 2000. Leaf feeding preferences in the monophagous saltbush flea beetle *Nesaecrepida darwini* (Coleoptera: Chrysomelidae). Bulletin van het Koninklijk Belgisch Instituut voor Natuurwetenschappen, Entomologie, 70: 255-258.

274. Cassola, F. , Roque-Albelo, L., K. Desender, 2000. Is the Endemic Galápagos Tiger Beetle Threatened with Extinction? Noticias de Galápagos, 61: 23-25.

275. DESENDER, K.,, VERDYCK, P, 2000. Genetic differentiation in the Galápagos caterpillar hunter *Calosoma granatense* (Coleoptera, Carabidae). In: BRANDMAYR, P et al. (ed.). Natural History and Applied Ecology of Carabid Beetles. Pensoft Publishers, Sofia, Moscow: 25-34.

276. DESENDER, K., SERRANO, J., VERDYCK, P., 2000. Genetic diversity and wing polymorphism in the saltmarsh beetle *Pogonus chalceus*: an Atlantic-Mediterranean comparison. In: BRANDMAYR, P et al. (ed.). Natural History and Applied Ecology of Carabid Beetles. Pensoft Publishers, Sofia, Moscow: 35-43.

277. THIJS, N., DESENDER, K., 2000. Ground beetles (Coleoptera, Carabidae and Cicindelidae) in the western part of Limburg (Flanders, Belgium). Bulletin de la Société royale belge d’Entomologie, 136: 35-39.

278. DESENDER, K.,, VERDYCK, P., 2001. Geographic scaling and genetic differentiation in two highly mobile European saltmarsh beetles. Belgian Journal of Zoology, 131: 29-40.

279. HENDRICKX, F., DESENDER, K., MAELFAIT, J.-P., 2001.Verspreidingspatronen en bedreigingen van de arthropodenfauna van het Schelde-estuarium. De Levende Natuur, 102: 68-69.

280. LOCK, K., DESENDER, K.,, JANSSEN, C.R., 2001. Effects of metal contamination on the activity and diversity of carabid beetles in an ancient Pb-Zn mining area at Plombières (Belgium). Entomologia Experimentalis et Applicata, 99:355-360.

281. BUSATO, E., DESENDER, K., P.M. GIACHINO, 2001. The larva of *Castrida granatense* (Géhin, 1885) (Coleoptera, Carabidae) from the Galápagos Islands (Ecuador). Bulletin van het Koninklijk Belgisch Instituut voor Natuurwetenschappen, Entomologie, 71:37-44.

282. DESENDER, K., 2001. Illustrated key to the genera of ground and tiger beetles of Galápagos (Coleoptera, Carabidae). Bulletin van het Koninklijk Belgisch Instituut voor Natuurwetenschappen, Entomologie, 71: 257-262.

283. PIGIERE, F., LENTACKER, A., DESENDER, K., GLEED-OWEN, C., VAN NEER, W., 2001. Epoque romaine. Antoing/Bruyelle: étude archéozoologique de la villa romaine de la <Haute Eloge>. Chronique de l’Archéologie wallonne, 9: 42-43.

284. SCHELVIS, J., DESENDER, K., LENTACKER, A., PIGIERE, F., 2001. V. Paléoenvironnement. 1. Etude des arthropodes. In: Liberchies. Vicus gallo-romain 4 (ed. BRULET, R., DEWERT, J.-P.,, VILVORDER, F.). Publications d’histoire de l’art et d’archéologie de l’UCL, pp. 411-414.

285. BOEKEN, M., DESENDER, K., DROST, B., VAN GIJZEN, T., KOESE, B., MUILWIJK, J., TURIN, H., R.J. VERMEULEN, 2002. De Loopkevers van Nederland, Vlaanderen (Coleoptera: Carabidae). Stichting Jeugdbondsuitgeverij, Utrecht: 212 pp.

286. DESENDER, K., CASALE, A., BAERT, L., MAELFAIT, JP, P. VERDYCK, 2002. *Calleida migratoria* (Coleoptera: Carabidae), a newly introduced ground beetle in Galapagos ? Coleopterists Bulletin, 56: 71-78.

287. DHUYVETTER, H.,GAUBLOMME, E., VERDYCK, P.,DESENDER, K., MONDOR-GENSON, G., RASPLUS, J.-Y., 2002. Isolation and characterization of microsatellite loci in Galápagos caterpillar hunters (Coleoptera, Carabidae, *Calosoma*). Molecular Ecology Notes, 2: 284-286.

288. ARNDT, E., DESENDER, K., 2002. Laboulbeniales (Ascomycota) on Carabidae (Insecta: Coleoptera) from the Galápagos Archipelago. Belgian Journal of Zoology, 132: 155-164.

289. DESENDER, K., ALDERWEIRELDT, M., 2002. Loopkevers in Oost-Vlaanderen. Bedreigde juweeltjes, onopvallende specialisten. Ommekeer : 10-15.

290. VERSTEIRT, V., DEKONINCK, W., DESENDER, K., GROOTAERT, P., 2002. Rediscovery of *Amara strenua* (ZIMMERMAN) in the ‘Uitkerkse polder’ area (Flanders, Belgium) (Coleoptera Carabidae). Bulletin de la Société royale belge d’Entomologie,138: 49-52.

291. DE BAKKER, D., MAELFAIT, J.-P., DESENDER, K., HENDRICKX, F., DE VOS, B, 2002. Regional variation in spider diversity of Flemish forest stands. In: TOFT, S., SCHARFF, N. (eds). European Arachnology 2000. Aarhus University Press, Aarhus, pp. 177-182.

292. DESENDER, K., ERVYNCK, A., 2002. Landschapsreconstructie op basis van de resten van loopkevers. In: COOREMANS, B., DESENDER, K., ERVYNCK, A.,, SCHELVIS, J. Onderzoek van plantaardige en dierlijke resten uit een Romeinse waterput van de vindplaats ‘Refuge’ te Sint-Andries, Brugge (prov. West-Vlaanderen): economie en ecologie. Archeologie in Vlaanderen VI, 1997/1998: 209-229.

293. DHUYVETTER, H.,GAUBLOMME, E., VERDYCK, P.,DESENDER, K., MONDOR-GENSON, G., RASPLUS, J.-Y., 2002. Isolation and characterization of microsatellite loci in the Galápagos Opuntia weevil Gerstaeckeria galapagoensis (Coleoptera, Curculionidae). Molecular Ecology Notes, 2: 475-477.

294. THYS, N., DESENDER, K., 2002. Loopkevers in het Vlaams natuurreservaat De Oudsberg (Meeuwen-Gruitrode): aanwijzingen voor het beheer. Natuur.Focus 1:137-142.

295. GAUBLOMME, E., DESENDER, K., VERDYCK, P., DHUYVETTER, H., J.-Y. RASPLUS, 2002. Non-destructive sampling for genetic studies on Carabus auronitens and Carabus problematicus: a study based on allozymes and microsatellites. In: Szyszko, J., den Boer, P.J.,, Bauer, Th. (eds.). How to protect or what we know about Carabid Beetles. Agric. Univ. Press, Warsaw: 337-344.

296. DESENDER, K., DE BAKKER, D., VERSTEIRT, V., B. DE VOS, 2002. A baseline study on forest ground beetle diversity and assemblages in Flanders (Belgium). In: Szyszko, J., den Boer, P.J.,, Bauer, Th. (eds.). How to protect or what we know about Carabid Beetles. Agric. Univ. Press, Warsaw: 237-245.

297. DESENDER, K., GAUBLOMME, E., F. HENDRICKX, P. VERDYCK, 2002. Population genetics and fluctuating asymmetry of an ancient forest ground beetle *Pterostichus cristatus*. In: Szyszko, J., den Boer, P.J.,, Bauer, Th. (eds.). How to protect or what we know about Carabid Beetles. Agric. Univ. Press, Warsaw: 273-286.

298. DESENDER, K., VERDYCK, P., GAUBLOMME, E., DHUYVETTER, H., J.-Y. RASPLUS, 2002. Extreme genetic differentiation and isolation by non-distance in *Carabus auronitens* in relation to forest historical ecology in Flanders (Belgium). In: Szyszko, J., den Boer, P.J.,, Bauer, Th. (eds.). How to protect or what we know about Carabid Beetles. Agric. Univ. Press, Warsaw: 227-235.

299. VERSTEIRT, V., DEKONINCK, W., DESENDER, K., GROOTAERT, P., 2002. Ground beetle communities as evaluation of reconverted arable land into heathland and dry oligotrophic grasslands (Flanders, Belgium). In: Szyszko, J., den Boer, P.J.,, Bauer, Th. (eds.). How to protect or what we know about Carabid Beetles. Agric. Univ. Press, Warsaw: 143-154.

300. VERDYCK, P., DESENDER, K., 2003. Mono- and oligophagous *Phyllotreta* (Coleoptera: Chrysomelidae) species: the relation between host plant range and genetic diversity. Belgian Journal of Zoology, 133: 71-76.

301. VERDYCK, P., DESENDER, K., DHUYVETTER, H., 2003. Genetic diversity of the phytophagous beetle *Docema darwini* Mutchler, 1925 (Coleoptera: Chrysomelidae), endemic to the Galápagos Islands. In: Furth, D.G. (ed.). Special topics in leaf beetle biology. Proc. 5th Int. Symp. on the Chrysomelidae. Pensoft Publ., Sofia Moscow, pp. 295-301.

302. GAUBLOMME, E., DHUYVETTER, H.,VERDYCK, P., MONDOR-GENSON, G., RASPLUS, J.-Y., DESENDER, K., 2003. Isolation and characterization of microsatellite loci in the ground beetle *Carabus problematicus* (Coleoptera, Carabidae). Molecular Ecology Notes, 3: 341-343.

303. DHUYVETTER, H.,, DESENDER, K., 2003. Isolation and characterization of microsatellite loci in the saltmarsh beetle *Pogonus chalceus* (Coleoptera, Carabidae). Molecular Ecology Notes, 3: 460-461.

304. DESENDER, K., HEIRBAUT, W., VERSTEIRT, V., DE BAKKER, D., 2003. Loopkevers in het Rodebos: Rode lijst-soorten, indicatoren voor oude bossen, dispersievermogen, populatiegenetica en habitatfragmentatie. Brakona Jaarboek 2001: 30-36.

305. HERMY, M. , L. DE KEERSMAEKER, K. VERHEYEN, P. ROOVERS, B. DE SOMVIELE, K. DESENDER,, K. VANDEKERKHOVE, 2003. Ecologische Functie van het bos in Vlaanderen. Bossenverklaring, Vlaamse Hoge Bosraad: 81-113.

306. DE BAKKER, D., K. DESENDER, P. VAN DE KERCKHOVE, MAELFAIT, J.-P., 2003. The spider fauna (Araneae) of ecological valuable alluvial forests: the case of the ‘Raspaille-Moerbeke-Karkoolbos’ complex (Eastern Flanders, Belgium). Bulletin de la Société royale belge d’Entomologie, 139: 194-203.

307. DESENDER, K., GAUBLOMME, E., VERDYCK, P., DHUYVETTER, H., DE VOS, B. 2004. Ecology and population genetics of *Carabus problematicus* in Flanders (Belgium): is forest history important? In: HONNAY, O., BOSSUYT, B., VERHEYEN, K., HERMY, M. (ed.). Forest Biodiversity: Lessons from History for Conservation. IUFRO res. Series. CAB International, Oxon, UK. 117-128.

308. DESENDER, K., ERVYNCK, A., 2004. Insectenresten uit de waterput. In: GELORINE, V.. Gemengde economische bedrijvigheid tijdens de meerfasige Ijzertijdbewoning te Zele: een palynologische bijdrage. Lunula, Archaeologica protohistorica XII, Aalst, p. 132.

309. DHUYVETTER, H., GAUBLOMME, E., DESENDER, K., 2004. Genetic differentiation and local adaptation in the saltmarsh beetle *Pogonus chalceus*:a comparison between allozyme and microsatellite loci. Molecular Ecology 13:1065-1074.

310. VERDYCK, P., DHUYVETTER, H., DESENDER, K., 2004. Genetic differentiation and population structure in *Metachroma labrale* Blair, 1933, a Galapagos leaf beetle (Chrysomelidae). In: JOLIVET, P., SANTIAGO-BLAY, J.A., M. SCHMITT., eds. New Developments in the Biology of Chrysomelidae, pp. 131-136. SPB Academic Publishing bv, The Hague, The Netherlands.

311. DESENDER, K., 2004. Loopkevers en zandloopkevers . In: Provoost, S., D. Bonte (red.). Levende duinen: een overzicht van de biodiversiteit aan de Vlaamse kust. Mededelingen van het Instituut voor Natuurbehoud 22, Brussel : 252-271.

312. POLLET, M., GROOTAERT, P., DESENDER, K., MAELFAIT, J.-P., 2004. Slankpootvliegen. In: Provoost, S., D. Bonte (red.). Levende duinen: een overzicht van de biodiversiteit aan de Vlaamse kust. Mededelingen van het Instituut voor Natuurbehoud 22, Brussel :236-251.

313. DEKONINCK W, DESENDER K, GROOTAERT P, MAELFAIT J-P, BAERT L, DE BAKKER D, T ADRIAENS, 2004. Observations of the ladybird *Platynaspis luteorubra* (Goeze) on motorway verges along the ring-road of Brussels, with comments on its habitat and host preference (Coleoptera Coccinellidae Chilocorinae). Bulletin De La Société Royale Belge d’Entomologie,140: 123-125.

314. DESENDER, K., GROOTAERT, P., DEKONINCK, W., BAERT, L., DE BAKKER, D., J.-P. MAELFAIT, 2004. Evaluatie van de natuurwaarde en het graslandbeheer van de bermen langs de noordelijke ring rond Brussel. Bulletin de la Société royale belge d’Entomologie,140: 126-139.

315. ANNAERT, R., COOREMANS, B., DESENDER, K., ERVYNCK, A., 2004.Een midden-Bronstijdwaterput en kuil uit de vroege Ijzertijd op de site Kapelleveld in Kontich (prov. Antwerpen). Archeologie in Vlaanderen VIII, 2001/2002: 79-103.

316. DE CLERCQ, W., BASTIAENS, K., DEFORCE, K., DESENDER, K., ERVYNCK, A., GELORINI, V., HANECA, K., LANGOHR, R., VAN PETEGHEM, A., 2004. Waarderend en preventief archeologisch onderzoek op de Axxes-locatie te Merelbeke (prov. Oost-Vlaanderen): een grafheuvel uit de Bronstijd en een nederzetting ui de Romeinse periode. Archeologie in Vlaanderen VIII, 2001/2002: 123-164.

317. VERHAERT, A., ANNAERT, R., LANGOHR, R., COOREMANS, B., GELORINI, V., BASTIAENS, K., DEFORCE, K., ERVYNCK, A.,, DESENDER, K., 2004. Een inheems-Romeinse begraafplaats te Klein-Ravels (gem. Ravels, prov. Antwerpen). Archeologie in Vlaanderen VIII, 2001/2002: 165-218.

318. DESENDER, K., SMALL, E., GAUBLOMME, E., VERDYCK, P., 2005. Rural-urban gradients and the population genetic structure of woodland ground beetles. Conservation Genetics 7: 1-12.

319. VERHEYEN, K., PIESSENS, K., DESENDER, K., VAN DYCK, H., VAN ELEGHEM, B., VERMEERSCH, G., VAN LANDUYT, W., MAES, D., 2005. Veranderingen in biodiversiteit van bos en heide door de eeuwen heen. Relaties tussen flora, fauna en landschapsdynamiek. Natuur.focus 4(2): 51-55.

320. DESENDER; K., 2005. A wingless intertidal ground beetle, new to the Belgian fauna, in the river IJzer estuary nature restoration site :*Bembidion nigropiceum Marsham, 1802.* Belgian Journal of Zoology, 135:89-90.

321. DESENDER, K.,2005. Theory versus reality: a review on the ecological and population genetic effects of forest fragmentation on wild organisms, with an emphasis on ground beetles. In: Lovei, G.L., Toft, S. (Eds.) Carabidology 2003. Proceedings of the 11th European Carabidologists’ Meeting, Århus, July 2003. Reports of the Danish Institute of Agricultural Sciences (DIAS Reports), Slagelse, Denmark: 49-72.

322. DESENDER, K., GAUBLOMME, E., DHUYVETTER, H., VERDYCK, P., 2005. Interaction between regional forest history, ecology and conservation genetics of *Carabus problematicus* in Flanders (Belgium). In: Lovei, G.L., Toft, S. (Eds.) Carabidology 2003. Proceedings of the 11th European Carabidologists’ Meeting, Århus, July 2003. Reports of the Danish Institute of Agricultural Sciences (DIAS Reports), Slagelse, Denmark: 73-87.

323. GAUBLOMME, E., DHUYVETTER, H., VERDYCK, P., DESENDER, K., 2005. Effects of urbanisation on carabid beetles in old beech forests. In: Lovei, G.L., Toft, S. (Eds.) Carabidology 2003. Proceedings of the 11th European Carabidologists’ Meeting, Århus, July 2003. Reports of the Danish Institute of Agricultural Sciences (DIAS Reports), Slagelse, Denmark: 111-123.

324. DESENDER, K., HONNAY, O.,, MAELFAIT, J.-P., 2005. Behoudsmaatregelen voor kleine en geïsoleerde populaties: verbinden of vergroten? Natuur.Focus 4(3): 95-100.

325. DESENDER, K., 2005. Ecologisch en genetisch onderzoek op loopkevers in de Makegemse bossen: even voorstellen. Groenlink apr-jun 2005: 1-7.

326. DHUYVETTER, H., GAUBLOMME, E., K. DESENDER, 2005. Bottlenecks, drift and differentiation: the fragmented population structure of the saltmarsh beetle *Pogonus chalceus.* Genetica 124: 167-177.

327. DHUYVETTER, H., GAUBLOMME, E., VERDYCK, P., K. DESENDER, 2005. Genetic Differentiation among populations of the saltmarsh beetle *Pogonus littoralis* (Coleoptera: Carabidae): a comparison between Atlantic and Mediterranean populations. Journal of Heredity 96:381-387.

328. GROOTAERT, P., DESENDER., K., VERSTEIRT, V., DEKONINCK, W., DE BAKKER, D., VAN DER WIJDEN, B., VERLINDE, R., 2005. Pilot study on tree canopy fogging in an ancient oak-beech plot of the Sonian forest (Brussels, Belgium). Bulletin de la Société royale belge d’Entomologie 141: 73-80.

329. GROOTAERT, P., DEKONINCK, W., DESENDER., K., 2005. Succession of dance fly fauna (Diptera: Empidoidea: Empididae, Hybotidae, Microphoridae) in ancient forests and afforested former agricultural land: a case-study in the “Voerstreek” (Belgium, Flanders): faunistics and new species for the Belgian fauna. Bulletin van het Koninklijk Belgisch Instituut voor Natuurwetenschappen, Entomologie, 75: 305-319.

330. HOFFMANN, M., ADAM, S., BAERT, L., BONTE, D., CHAVATTE, N., CLAUS, R., DE BELDER, W., DE FRÉ, B., DEGRAER, S., DE GROOTE, D., DEKONINCK, W., DESENDER, K., DEVOS, K., ENGLEDOW, H., GROOTAERT, P., HARDIES, N., LELIAERT, F., MAELFAIT, J.-P.,MONBALIU, J., POLLET, M., PROVOOST, S., STICHELMANS, E., TOORMAN, E., VAN NIEUWENHUYSE, H., VERCRUYSSE, E., VINCX, M.,, WITTOECK, J., 2005. Integrated monitoring of nature restoration along ecotones, the example of the Yser Estuary. In: Herrier J.-L., J. Mees, A. Salman, J. Seys, H. Van Nieuwenhuyse, I. Dobbelaere (Eds). Proceedings ‘Dunes and Estuaries 2005’ – International Conference on Nature Restoration Practices in European Coastal Habitats, Koksijde, Belgium, 19-23 September 2005. VLIZ Special Publication 19, xiv + 685pp. p. 191-208.

331. DESENDER, K., BAERT, L.,, MAELFAIT, J.-P., 2005. Evaluation of the effects of recent nature development measures in the Yser Estuary on ground beetle and spider assemblages. In: Herrier J.-L., J. Mees, A. Salman, J. Seys, H. Van Nieuwenhuyse, I. Dobbelaere (Eds). Proceedings ‘Dunes and Estuaries 2005’ – International Conference on Nature Restoration Practices in European Coastal Habitats, Koksijde, Belgium, 19-23 September 2005. VLIZ Special Publication 19, xiv + 685pp. p. 595-596.

332. DESENDER, K., 2005. Ground beetle diversity monitoring and the evaluation of a recent nature restoration project: A long-term case study in dune and salt marsh habitats along the Belgian coast. In: Serrano, J., J. Gomez-Zurita, C. Ruiz (Eds). XII European Carabidologists Meeting, ‘Ground beetles as a key group for biodiversity conservation studies in Europe’.Nausicaa Edicion Electronica, Murcia, Spain: 29-38.

333. DESENDER, K., DEKONINCK, W., P. GROOTAERT, 2005. Diversity and assemblages of carabid beetles in ancient forests and afforested former agricultural land. Bulletin van het Koninklijk Belgisch Instituut voor Natuurwetenschappen, Entomologie 75: 253-265.

334. DEKONINCK, W., DESENDER, K., GROOTAERT, P., J.-P. MAELFAIT, 2005. Afforestation, plantation and extensive grazing on formerly agricultural sites and the influences on different arthropod-groups: a case-study in the Voeren-region. Bulletin van het Koninklijk Belgisch Instituut voor Natuurwetenschappen, Entomologie 75: 221-234.

335. DEKONINCK, W., MAELFAIT J.-P., GROOTAERT, P., K. DESENDER, 2005. Comparative study of the terrestrial isopod faunas of ancient forests and afforested former agricultural fields. Bulletin van het Koninklijk Belgisch Instituut voor Natuurwetenschappen, Entomologie 75: 271-279.

335b.GROOTAERT, P., DEKONINCK, W., K. DESENDER, 2005. Succession of dance fly fauna (Diptera: Empidoidea: Empididae, Hybotidae, Microphoridae) in ancient forests and afforested former agricultural land: a case-study in the “Voerstreek” (Belgium, Flanders): faunistics and new species for the Belgian fauna. Bulletin van het Koninklijk Belgisch Instituut voor Natuurwetenschappen, Entomologie 75: 305-319.

336. VAN IMPE, L., IN ‘t VEN, I., DE PAEPE, P., ERVYNCK, A., K. DESENDER, 2005. Invading tribes, advancing forests. A witness of the decline of economic activity in Flanders, circa 200 AD. Studien zur Sachsenforschung 15: 287-305.

337. DESENDER, K., 2006. Wanneer bosloopkevers op stap gaan in de Makegemse bossen… Een verhaal over variatie in levenscycli, reproductietiming en biologische aanpassingen. Groenlink apr-jun 2006:19-32.

338. DESENDER, K.R.C., S.B PECK, 2006. Family Carabidae, The Predaceous Ground Beetles and Tiger Beetles. In: PECK, S.B. The Beetles of the Galápagos Islands, Ecuador; Evolution, Ecology, and Diversity (Insecta: Coleoptera). NRC Research Press, Ottawa, Ontario, Canada: p. 101-117.

339. VAN NEER, W., HAMILTON-DYER, S., CAPPERS, R., DESENDER, K., ERVYNCK, A. 2006. The Roman trade in salted Nilotic fish products: some examples from Egypt. Documenta Archaeobiologiae 4: 173-188.

340. DESENDER, K., BAERT, L., MAELFAIT, JP, P. VERDYCK, 2006. Effects of the feral goat population explosion on Alcedo volcano (Isabela, Galapagos) between 1986 and 1996. Galápagos Research 64: 2-7.

341. DESENDER, K., BAERT, L., JP MAELFAIT, 2006. Evaluation of recent nature development measures in the river IJzer estuary and long-term ground beetle and spider monitoring (Coleoptera, Carabidae; Araneida). Bulletin van het Koninklijk Belgisch Instituut voor Natuurwetenschappen, Entomologie 76: 103-122.

342. DESENDER, K., DHUYVETTER, H., DRUMONT, A., N. WARZEE, 2006. Forest ground beetle assemblages and population genetics in the Wellin district (Ardennes, Belgium): a forest historical approach. Bulletin van het Koninklijk Belgisch Instituut voor Natuurwetenschappen, Entomologie 76: 123-133.

343. DUFRENE, M., DESENDER, K. 2006. L’érosion de la biodiversité: les carabides,Dossier scientifique réalisé dans le cadre de l’élaboration du Rapport analytique 2006-2007 sur l’état de l’environnement Wallon. Centre de la Recherche de la Nature, des Forêts et du Bois – Gembloux et Institut royal des Sciences naturelles de Belgique – Bruxelles. 28 pp.

344. DHUYVETTER, H., HENDRICKX, F., GAUBLOMME, E., K. DESENDER, 2007. Differentiation between two salt marsh beetle ecotypes: evidence for ongoing speciation. Evolution 61: 184-193.

345. HELSEN, P.,VERDYCK, P.,TYE, A., DESENDER, K., VAN HOUTTE, N., S. VAN DONGEN, 2007. Isolation and characterization of polymorphic microsatellite markers in Galapagos prickly pear (*Opuntia*) cactus species. Molecular Ecology Notes 3.

346. DESENDER, K., J.P. MAELFAIT, 2007. Langetermijnonderzoek op loopkevers (coleoptera, carabidae) in het walenbos. Brakona Jaarboek2005: 96-105.

347. DHUYVETTER, H. MAELFAIT, J.-P., K. DESENDER, 2007. Inter- and intraspecific genetic and morphological variation in a sibling pair of carabid species. Saline Systems 3, 4.

348. DUFRENE M., DESENDER K., 2007. Rapport analytique sur l’état de l’environnement Wallon 2006-2007. L’érosion de la biodiversité: les carabides: 596-597.

349. LENTACKER, A., VAN NEER, W., ERVYNCK, A., K. DESENDER, 2007. Het Steen en de burgers. Onderzoek van de laatmiddeleeuwse gevangenis van Mechelen. De ecologische gegevens: de dierlijke resten: 133-154. Drukkerij Artoos, Kampenhout. Uitgave stad Mechelen.

350. VAN NEER, W., W. WOUTERS, M.-H. RUTSCHOWSCAYA, A. DELATTRE, D. DIXNEUF, K. DESENDER, J. POBLOME, 2007. Salted fish products from the coptic monastery at Bawit, Egypt: evidence from the bones and texts. Proceedings of the 13th meeting of the Icaz fish remains working group: 147-159.

351. DESENDER, K., MAELFAIT, J.-P., BAERT, L., 2007. Ground beetles as ‘early warning-indicators’ in restored salt marshes and dune slacks. Coastline Reports 7: 25-39.

352. MAELFAIT, J.-P., DESENDER, K., BAERT, L., 2007. Colonisation and source-sink dynamics in spiders and ground beetles after dry dune habitat restoration along the belgian coast. Coastline Reports 7: 41-52.

353. DESENDER, K., DEKONINCK, W., K. SMETS, 2007. First record of *Abax carinatus* in Flanders and notes on an inventory of ground beetles in the forest reserve Jagersborg (Maaseik). Bulletin de la Société royale belge d’Entomologie 143 : 15-22.

354. DEKONINCK, W., DESENDER K., P. GROOTAERT, 2008. Establishment of ant communities in forests established on former agricultural fields: colonisation and 25 years of management are not enough (Formicidae: Hymenoptera). European Journal of Entomology, 105(4): 681-689.

355. DESENDER, K., DEKONINCK, W., D. MAES, 2008. An updated Red List of the ground and tiger beetles (Coleoptera, Carabidae) in Flanders (Belgium). Bulletin van het Koninklijk Belgisch Instituut voor Natuurwetenschappen, Entomologie, 78: 113-131.

356. DESENDER, K., DEKONINCK, W., MAES, D. m.m.v. CREVECOEUR, L., DUFRENE, M., JACOBS, M., LAMBRECHTS, J., POLLET, M., STASSEN, E., THYS, N., 2008. Een nieuwe verspreidingsatlas van de loopkevers en zandkloopkevers (Carabidae) in België. Rapporten van het Instituut voor Natuur- en Bosonderzoek 2008 (INBO.R.2008.13). Instituut voor Natuur- en Bosonderzoek, Koninklijk Belgisch Instituut voor Natuurwetenschappen, Brussel.

357. DESENDER, K., 2007. Genetic study of salt marsh ground beetles of the Coto Doñana. Oficina de coordinación de la investigación.Estación Biológica de Doñana, CSIC, FICHA DE RESULTADOS Nº: 9/2007.

### Additions

De Wolf,H., Thys, P., Desender, K., Th. Backeljau, submitted 2006. Lack of allozyme variation in the pulmonate mollusc, *Myosotella myosotis* (Draparnaud, 1801), from the Western Scheldt estuary (The Netherlands). Marine Biology Research

Matern, A., Desender, K, Dress, C., Gaublomme, E., Paill, W.,, Assmann, T., 2008. Genetic diversity and population structure of the endangered insect species *Carabus variolosus* in its western distribution range: Implications for conservation. *Conservation Genetics* DOI:10.1007/s10592-008-9606-1

Gaublomme, E., Hendrickx, F., Dhuyvetter, H., Desender, K, 2008. The effects of forest patch size and matrix type on changes in carabid beetle assemblages in an urbanized landscape. *Biological Conservation* 141: 2585-2596

### Unpublished work and internal reports

1. DESENDER, K., 1978. Inventarisatie en oecologie van fauna-elementen in het natuurreservaat ‘De Blankaart’ (Prov. West-Vlaanderen). Licentiaatsverhandeling R.U.Gent, 185 pp.

2. DESENDER, K., 1979. Methodologisch en oecologisch onderzoek van Carabidae in graslandbiotopen. Verslag I.W.O.N.L., 1e werkjaar, 24 pp.

3. DESENDER, K., 1980. Oecologisch onderzoek van Carabidae in graslandbiotopen. Verslag N.F.W.O., 1e werkjaar, 25 pp.

4. MAELFAIT, J.-P., DESENDER, K., D’HULSTER, M., VANHERCKE, L., 1980. Beschrijvende en functionele oecologie van coprophage, sapronecrophage en carnivore kevers van verschillende oecosystemen. F.K.F.O.-project 2-9002-79: verslag voor het werkingsjaar 1979, 80 pp.

5. DESENDER, K., 1981. Oecologisch Onderzoek van Carabidae in graslandbiotopen. Verslag N.F.W.O., 2e werkjaar, 37 pp.

6. MAELFAIT, J.-P., DESENDER, K., D’HULSTER, M., VANHERCKE, L., 1981. F.K.F.O.-project 2-9002-79: Verslag voor het werkingsjaar 1980, 10 pp.

7. MAELFAIT, J.-P., DESENDER, K., D’HULSTER, M., VANHERCKE, L., 1982. F.K.F.O.-project 2-9002-79: Verslag voor het werkingsjaar 1981, 8 pp.

8. DESENDER, K., 1984. Dispersie en geografische verspreiding van organismen. Stencil, R.U. Gent, 12 pp.

9. DESENDER, K., 1987. Bemonsteringsmethodologie, Levenscyclus, en Evolutionaire Ecologie van het Dispersievermogen bij Loopkevers (Coleoptera, Carabidae). Doctoraatsverhandeling, R.U. Gent. Deel I: Tekst (346 pp); Deel II: Tabellen en Figuren; Deel III: Bijlagen.

10. DESENDER, K., 1988. Etudes des caractéristiques des populations animales. Cursus ‘Post-graduation en écologie animale’, Algiers, 34 pp.

11. DESENDER, K., 1988. Dispersion et distribution géographique des organismes. Cursus ‘Post-graduation en écologie animale’, Algiers, 12 pp.

12. DESENDER, K., ALDERWEIRELDT, M., 1988. Invloed van het gebruik van een terraverde-mengsel op de pedofauna (Burkina Faso, 1988). Rapport van het onderzoeksproject ‘Invloed van Hydroabsorbentia, meststoffen en groeistimulatoren op de beplanting van droge of gedegradeerde bodems en hun pedofauna’. Onderzoeksfonds R.U.Gent, 1988, 7 pp.

13. DESENDER, K., ALDERWEIRELDT, M., DE KEER, R., POLLET, M., 1988. Ecologisch onderzoek naar het voorkomen, de funktie en impakt van spinnen en loopkevers in akkers, graasweiden en hun randzones: implikaties voor biologische bestrijding en natuurbehoud. Rapport van het project 01160188 OZF RUG, 4 pp + 11 bijlagen.

14. DESENDER, K., ALDERWEIRELDT, M., POLLET, M., BAGUETTE, M., DUFRENE, M., LOREAU, M., RASMONT, P., GASPAR, Ch., 1990. Ecologie, kartografie en faunistiek van loopkevers (Coleoptera, Carabidae) in België en Noord-Frankrijk. Eindverslag FKFO-project 2.9008.89, 12 pp.

15. MAELFAIT, J.-P., DESENDER, K., ALDERWEIRELDT, M., 1991. Ecologie, kartografie en faunistiek van loopkevers (Coleoptera, Carabidae) in België en Noord-Frankrijk. Vorderingsverslag FKFO-projekt 2.9014.91, 1e werkjaar, 7 pp + 1 bijlage.

16. ALDERWEIRELDT, M., DESENDER, K., MAELFAIT, J.-P., 1992. Ecologie, kartografie en faunistiek van loopkevers (Coleoptera, Carabidae) in België en Noord-Frankrijk. Vorderingsverslag FKFO-projekt 2.9014.91, eindverslag, 8 pp.

17. MAES, D., DESENDER, K., VAN KERCKVOORDE, M., 1994. Een gedokumenteerde rode lijst van de zandloop- en loopkevers van Vlaanderen (Coleoptera: Cicindelidae, Carabidae). Rapport in opdracht van het Instituut voor Natuurbehoud, 198 pp.

18. MAELFAIT, J.-P., DE BRUYN, L., DE MEESTER, L., BACKELJAU, Th.,, DESENDER, K., 1996. Populatiegenetisch onderzoek aan ongewervelden, met toepassingen voor het natuurbehoud. Vorderingsverslag FKFO G.2128.94

19. DE MEESTER, L., VOLCKAERT, F., MAELFAIT, J.-P., VERHAGEN, R., BACKELJAU, Th., DESENDER, K., VERDYCK, P.,, TRIEST, L., 1997. Genetisch-ecologisch onderzoek ten behoeve van het natuurbehoud in Vlaanderen. Aanvangsverslag Project AMINAL/VLINA 96/01, 29 pp.

20. De Bakker, D., Desender, K., P. Grootaert, 2000. Determinatie en bioindicatie van bosgebonden ongewervelden. 1. Bioindicatie van standplaatsvariabelen. Onderzoeksopdracht B&G/29/98, AMINAL. Rapport ENT.2000.01, KBIN, Brussel, 146 pp.

21. Desender, K, De Bakker, D., P. Grootaert, 2000. Determinatie en bioindicatie van bosgebonden ongewervelden. 2. Platenatlas van indicatorsoorten (loopkevers, spinnen en vliegen). Onderzoeksopdracht B&G/29/98, AMINAL. Rapport ENT.2000.02, KBIN, Brussel, inleiding + 10 platen.

22. VERSTEIRT, V., DESENDER, K., GEUDENS, G., P. GROOTAERT, 2000. . Determinatie en bioindicatie van bosgebonden ongewervelden.3. Ecologische standplaatskarakterisatie van bossen aan de hand van keverfauna (Coleoptera). 4. Verkennend onderzoek naar de potentiële waarde van integrale bosreservaten voor het behoud van xylobionte arthropoden. Rapport ENT.2000.03 en ENT.2000.04, Onderzoeksopdracht B&G/29/98,193 pp.

23. De Meester, L., F. Volckaert, E. Audenaert, E. Michiels, J. Vanoverbeke, S. Declercq, B. Hellemans, J.-P. Maelfait, B. Neyrinck, R. Verhagen, L. De Bruyn, Th. Backeljau, K. Desender, P. Verdyck, L. Triest, B. De Greef, 2000. *Genetisch-ecologisch onderzoek ten behoeve van het natuurbehoud*. Vlaams Impulsprogramma Natuurontwikkeling. Eindrapport VLINA96/01.

24. De Bakker, D., Desender, K., P. Grootaert, 2000. Determinatie en bioindicatie van bosgebonden ongewervelden. 1. Bioindicatie van standplaatsvariabelen. Onderzoeksopdracht B&G/29/98, AMINAL. Rapport ENT.2000.01, KBIN, Brussel, 146 pp.

25. Desender, K, De Bakker, D., P. Grootaert, 2000. Determinatie en bioindicatie van bosgebonden ongewervelden. 2. Platenatlas van indicatorsoorten (loopkevers, spinnen en vliegen). Onderzoeksopdracht B&G/29/98, AMINAL. Rapport ENT.2000.02, KBIN, Brussel, inleiding + 10 platen.

26. VERSTEIRT, V., DESENDER, K., GEUDENS, G., P. GROOTAERT, 2000. Determinatie en bioindicatie van bosgebonden ongewervelden.3. Ecologische standplaatskarakterisatie van bossen aan de hand van keverfauna (Coleoptera). 4. Verkennend onderzoek naar de potentiële waarde van integrale bosreservaten voor het behoud van xylobionte arthropoden. Rapport ENT.2000.03 en ENT.2000.04, Onderzoeksopdracht B&G/29/98, 193 pp.

27. Desender, K., p. Verdyck, v., E. GAUBLOMME, B. DE VOS, N. ROGIERS, L. VANHOUTTE, 2000. Historische en recente bosontwikkeling in Vlaanderen: habitatkarakterisatie en niet-destructief conservatiegenetisch onderzoek bij loopkevers. Aanvangsverslag VLINA/0015, 8 pp.

28. DE BAKKER, D.; DESENDER, K.; GROOTAERT, P., BAERT, L. , 2001. Inventarisatie en determinatie van ongewervelden als ecologische indicatoren in Vlaamse integrale bosreservaten. 1. Het belang van integrale bosreservaten voor arboricole en bodembewonende spinnen en loopkevers. Rapport ENT.2001.01. Onderzoeksopdracht B&G/19/99: 90pp + bijlagen.

29. DESENDER, K.; DE BAKKER, D., VAN DE KERCKHOVE, P. , 2001. Inventarisatie en determinatie van ongewervelden als ecologische indicatoren in Vlaamse integrale bosreservaten. 2. Casestudie naar de invloed van boshistoriek, bosfragmentatie en bosexploitatie op de spinnen- en loopkeverfauna van ecologisch waardevolle alluviale bossen. Rapport ENT.2001.03. Onderzoeksopdracht B&G/18/99: 98pp + 26 bijlagen.

30. HEIRBAUT, W.; DESENDER, K.; DE BAKKER, D., GROOTAERT, P. , 2001. Inventarisatie en determinatie van ongewervelden als ecologische indicatoren in Vlaamse integrale bosreservaten. Inventarisatie en evaluatie van bodembewonende en xylobionte arthropoden in integrale bosreservaten. Partim xylobionte arthropoden. Rapport ENT.2001.04. Onderzoeksopdracht B&G/18/99.

31. DESENDER, K., VERSTEIRT, V., BAETENS, J. , 2001. Carabidae. In: BAETENS, J., P. GROOTAERT. De betekenis van lijn- en puntvormige rietvegetaties voor semi-terrestrische ongewervelden van moerashabitats. Studie in opdracht van het Vlaams Ministerie van Leefmilieu en Landbouw en het Instituut voor Natuurbehoud. Rapport ENT.2001.06: 32-46.

32. De Bakker, D., Desender, K., HEIRBAUT, W., , 2002. Inventarisatie en determinatie van ongewervelden als ecologische indicatoren in Vlaamse integrale bosreservaten. 4. Het belang van integrale bosreservaten voor arboricole en bodembewonende spinnen en loopkevers. Rapport ENT.2001.05. Onderzoeksopdracht B&G/19/99: 144 pp. + 31 bijlagen.

33. Desender, K., p. Verdyck, v., E. GAUBLOMME, B. DE VOS, N. ROGIERS, L. VANHOUTTE, P. QUATAERT, 2002. Historische en recente bosontwikkeling in Vlaanderen: habitatkarakterisatie en niet-destructief conservatiegenetisch onderzoek bij loopkevers. Eindverslag VLINA/0015

34. DESENDER K, DEKONINCK W, BAERT L, GROOTAERT P, J-P MAELFAIT, 2004. “In de ban van de ring’. Inventarisatie van een aantal invertebratengroepen op de bermen, de taluds en de restgronden van de R0 (Ring van Brussel en een voorstel tot monitoring. Rapport ENT.2004.01. i.o.v. AMINAL, cel NTMB, KBIN, Brussel, 61 pp.

35. DESENDER, K., DE BAKKER, D., DEKONINCK, W., 2004. Verkennende inventarisatie van loopkevers, spinnen, en mieren in het provinciaal Domein ‘de Halve Maan’ (Diest). Resultaten van een kortetermijnbemonstering in 6 habitatten met vermelding van bijkomende gegevens van enkele andere insectengroepen. Rapport Onderzoeksopdracht Econnection. 17pp. + bijlagen.

36. DESENDER K, BAERT L,, J-P MAELFAIT, 2004.Evaluatie van recente natuurontwikkelingsmaatregelen aan de IJzermonding en langetermijnmonitoring van loopkevers en spinnen. Eindrapport MONAY-project. 20 pp. + bijlagen.

37. DESENDER, K., BAERT, L., J.-P. MAELFAIT, 2005. MONAIJ. Evaluatie van recente natuurontwikkelingsmaatregelen aan de IJzermonding en langetermijnmonitoring van loopkevers en spinnen. In; Hoffmann, M. :Evaluatie van de impact van recente natuurontwikkelingsmaatregelen aan de Ijzermonding: pp 125-142 + 10pp bijlagen 1-6.

38. DEKONINCK, W., DESENDER, K., P. GROOTAERT, 2005. Faunistische evaluatie en vergelijking van bosuitbreiding via bebossing, spontane verbossing, en extensieve begrazing van open terreinen: een case-study in de Voerstreek. Niet-technische en niet-wetenschappelijke samenvatting. 14 pp.

39. DEKONINCK, W., DESENDER, K., P. GROOTAERT, 2005. Faunistische evaluatie en vergelijking van bosuitbreiding via bebossing, spontane verbossing, en extensieve begrazing van open terreinen: een case-study in de Voerstreek. AMINAL/B&G/30/2002 project 3. Rapport ENT.2005.01. 190pp.

40. DESENDER, K., W. DEKONINCK, 2005. Loopkevers en zandloopkevers – Carabidae. Faunistische evaluatie en vergelijking van bosuitbreiding via bebossing, spontane verbossing, en extensieve begrazing van open terreinen: een case-study in de Voerstreek. AMINAL/B&G/30/2002 project 3. Rapport ENT.2005.01.: 40-60.

41. DESENDER, K., BAERT, L., J.-P. MAELFAIT, 2005. MONAIJ. Evaluatie van recente natuurontwikkelingsmaatregelen aan de IJzermonding en langetermijnmonitoring van loopkevers en spinnen. In; Hoffmann, M.: Evaluatie van de impact van recente natuurontwikkelingsmaatregelen aan de Ijzermonding: pp 125-142 + 10pp bijlagen 1-6.

42. DUFRÊNE, M., K. DESENDER, 2006. EEW 06 - Analyse Carabides. Etat de l’environnement Wallon, 26 pp.

43. DESENDER, K., DEKONINCK, W., 2006. ‘in de ban van de ring’: diversiteit en kwaliteit van een aantal invertebratengroepen op de bermen, taluds en restgronden van de R0 (Ring van Brussel). Brussel, Cursusmap ‘Natuurtechnische Milieubouw’, Themadag Bermbeheer langs wegen, 19 mei 2006: 4 pp.

44. DESENDER, K., BAERT, L., J.-P. MAELFAIT, 2006. MONAIJ. Evaluatie van recente natuurontwikkelingsmaatregelen aan de IJzermonding en langetermijnmonitoring van loopkevers en spinnen. In; Hoffmann, M.: Evaluatie van de impact van recente natuurontwikkelingsmaatregelen aan de Ijzermonding: pp 125-142 + 10pp bijlagen 1-6.

45. DESENDER, K., BAERT, L., MAELFAIT, J.-P., F. HENDRICKX, 2006. Biologische responsvariabelen: Arthropoda van schor en duin. In: Hoffmann, M. (ed.) MONAIJ: Monitoring Natuurherstel IJzermonding 2001-2005. Eindrapport onderzoeksopdracht AN.GKB/2001/nr. 1: 215-232 + 12 bijlagen

46. Dufrêne, M., Desender, K. 2006. Rapport analytique sur l’état de l’environnement Wallon 2006-2007. L’érosion de la biodiversité: les carabides, 28 pp.

47. Dekoninck, W., Desender, K., 2007.Een nieuwe verspreidingsatlas en rode lijst van de loopkevers van Vlaanderen (Coleoptera, Carabidae). Eindverslag INBO/TWOL-2006-01, 33pp.

